# Mouse genome-wide association studies and systems genetics uncover the genetic architecture associated with hepatic pharmacokinetic and pharmacodynamic properties of a constrained ethyl antisense oligonucleotide targeting *Malat1*

**DOI:** 10.1371/journal.pgen.1007732

**Published:** 2018-10-29

**Authors:** Elaine Pirie, Shayoni Ray, Calvin Pan, Wuxia Fu, Andrew F. Powers, Danielle Polikoff, Colton M. Miller, Katrina M. Kudrna, Edward N. Harris, Aldons J. Lusis, Rosanne M. Crooke, Richard G. Lee

**Affiliations:** 1 Cardiovascular Antisense Drug Discovery Group, Ionis Pharmaceuticals, Carlsbad, California, United States of America; 2 Department of Human Genetics, University of California, Los Angeles, Los Angeles, California, United States of America; 3 Exploratory Drug Discovery Group, Ionis Pharmaceuticals, Carlsbad, California, United States of America; 4 Department of Biochemistry, University of Nebraska-Lincoln, Lincoln, NE, United States of America; Stanford University School of Medicine, UNITED STATES

## Abstract

Antisense oligonucleotides (ASOs) have demonstrated variation of efficacy in patient populations. This has prompted our investigation into the contribution of genetic architecture to ASO pharmacokinetics (PK) and pharmacodynamics (PD). Genome wide association (GWA) and transcriptomic analysis in a hybrid mouse diversity panel (HMDP) were used to identify and validate novel genes involved in the uptake and efficacy of a single dose of a Malat1 constrained ethyl (cEt) modified ASO. The GWA of the HMDP identified two significant associations on chromosomes 4 and 10 with hepatic Malat1 ASO concentrations. Stabilin 2 (*Stab2*) and vesicle associated membrane protein 3 (*Vamp3)* were identified by cis-eQTL analysis. HMDP strains with lower *Stab2* expression and *Stab2* KO mice displayed significantly lower PK than strains with higher *Stab2* expression and the wild type (WT) animals respectively, confirming the role of *Stab2* in regulating hepatic Malat1 ASO uptake. GWA examining ASO efficacy uncovered three loci associated with Malat1 potency: Small Subunit Processome Component (*Utp11l*) on chromosome 4, Rho associated coiled-coil containing protein kinase 2 (*Rock2*) and Aci-reductone dioxygenase (*Adi1)* on chromosome 12. Our results demonstrate the utility of mouse GWAS using the HMDP in detecting genes capable of impacting the uptake of ASOs, and identifies genes critical for the activity of ASOs *in vivo*.

## Author summary

Previous work in the clinic has clearly demonstrated differential patient response to antisense oligonucleotide (ASO) drugs. However, to date there has been no systematic evaluation of genes associated with this response *in vivo*. In this study, we utilized an advanced genetic methodology in mice to identify genes involved with the heterogeneity in both accumulation and potency of an ASO targeting metastasis associated lung adenocarcinoma transcript 1 (Malat1) in liver. Detailed analysis of ASO functionality in livers from 100 genetically distinct strains of inbred mice treated with either Malat1 or control ASO led to the selection of specific genetic regions associated with variation in ASO uptake and potency. Specifically, we identified regions on chromosomes 4 and 10 which highlighted two genes associated with variations in hepatic drug accumulation. Further, we established three regions on chromosome 4 and 12 linked to three genes associated with variability in hepatic ASO efficacy. We carried out additional functional validation of the isolated genes in mouse models and cell lines and confirmed that this methodology can be used to identify genes affecting ASO drug response. These results are particularly important for the design of antisense drugs with improved efficacy, safety, and tolerability.

## Introduction

Antisense oligonucleotides (ASOs) are highly selective and potent therapeutic agents which has proven effective in treating a variety of disease states including cancer, viral infection and cardio-metabolic, inflammatory and neurological diseases [1–7]. Antisense technology uses short synthetic (12–24 mers), chemically modified DNA-like oligonucleotides to alter the intermediary metabolism of RNAs. The most widely exploited mechanism of ASO functionality is the degradation of complementary mRNA utilizing ribonuclease H1 (RNase H1), thereby preventing the translation of the associated protein(s) [8]. The evolution of this technology over the past 30 years has led to the development of a variety of ASO modifications resulting in greater stability, potency, affinity, and reduced toxicity [9, 10].

While these therapeutic agents have proven to be effective against disease in the clinic, significant variability in ASO response has been reported in several of the drugs [11, 12]. For example Kynamro, a second-generation 2´ methoxyethyl ASO targeting human apoB, has been approved by the FDA for use as an adjunct with first-line therapies to reduce apoB and total cholesterol in homozygous familial hypercholesterolemia (FH) patients. In Phase 3 trials, FH patients who were administered 200 mg/week of the drug observed reductions in plasma LDL-C levels ranging from 2% to –82% [1]. Differences in observed efficacy is not unique to the ASO platform: genetic make-up may account for 20%-95% of overall variability of therapeutic agents in general [13]. Importantly, previous research has indicated that pathways leading to ASO tissue accumulation and pathways driving ASO activity may be segregated due to the presence of ASO ‘sinks’, trapping ASO within endocytotic compartments [14, 15]. To date, no *in vivo* systematic interrogation of the role of genetic architecture on ASO accumulation and potency has been performed.

Genome-wide association studies (GWAS) provide 1) hypothesis-free mapping and 2) the identification of causal variants within the genome that contribute to the variability of a complex trait. The utilization of this system has allowed the detection of single-nucleotide polymorphisms (SNPs) leading to novel mechanistic insights and the identification of hundreds of genes potentially causal for human pathophysiological states such as diabetes, cancer and numerous cardiovascular diseases [16, 17]. Association studies in mouse models provide multiple advantages over large scale analyses in human populations, including cost effectiveness, reproducibility of results and the reduced impact of environmental factors. The hybrid mouse diversity panel (HMDP) is one such resource. It consists of over 100 genetically unique inbred mouse strains– 30 “classical inbred” strains, in addition to over 70 “recombinant inbred” strains. The recombinant inbred strains were derived from the F1 crosses of eight inbred “founder strains” and are able to offer important insights into genetically-derived differences in phenotype among inbred mice. The strains making up the HMDP are genotyped at 140,000 high quality SNPs [18–20], with sufficient power to detect traits contributing to 10% of overall phenotypic variance. GWAS carried out with the HMDP have identified significant SNPs in a wide variety of phenotype measurements such as NAFLD, bone mineral density, insulin resistance, obesity and heart failure [21–25].

To better understand the genetic variability and genomic variants that are associated with ASO uptake and potency, we employed GWAS and transcriptomic analysis of the HMDP using a generation 2.5 2´, 4´- constrained 2´-O- ethyl (cEt) ASO [9] targeting the murine long noncoding RNA metastasis associated lung adenocarcinoma transcript 1 (*Malat1*). Following a single dose of this ASO, both hepatic *Malat1* expression and tissue accumulation were evaluated in 100 HMDP strains. Significant intra-strain variability was observed in both hepatic Malat1 ASO potency and accumulation. Using Factored Spectrally Transformed Linear Mixed Model (FastLMM) [26, 27], we identified two loci on chromosomes 4 and 10 associated with hepatic Malat1 ASO accumulation. Systems genetic analysis determined these loci contributed to expression of Stabilin 2 (*Stab2*) on chromosome 10 and vesicle associated membrane protein 3 (*Vamp3*) on chromosome 4. Additionally, we identified three loci, two on chromosome 12 and one on chromosome 4, associated with variation in hepatic Malat1 ASO potency. Rho associated coiled-coil containing protein kinase 2 (*Rock2*) and Aci-reductone dioxygenase (*Adi1*) were identified as high confidence candidate genes regulating ASO PD in chromosome 12, while UTP11 small subunit processome component (*Utp11l*) was identified on chromosome 4. Additional *in vitro* studies validated *Rock2* contributions to the hepatic potency of the Malat1 ASO. Our results demonstrate that genetic variation impacts ASO PK/PD and validates the use of the HMDP GWAS in advancing our understanding of the molecular mechanisms that contribute to ASO biology.

## Results

### Genome-wide association analysis of hepatic ASO accumulation

**Fig 1:**
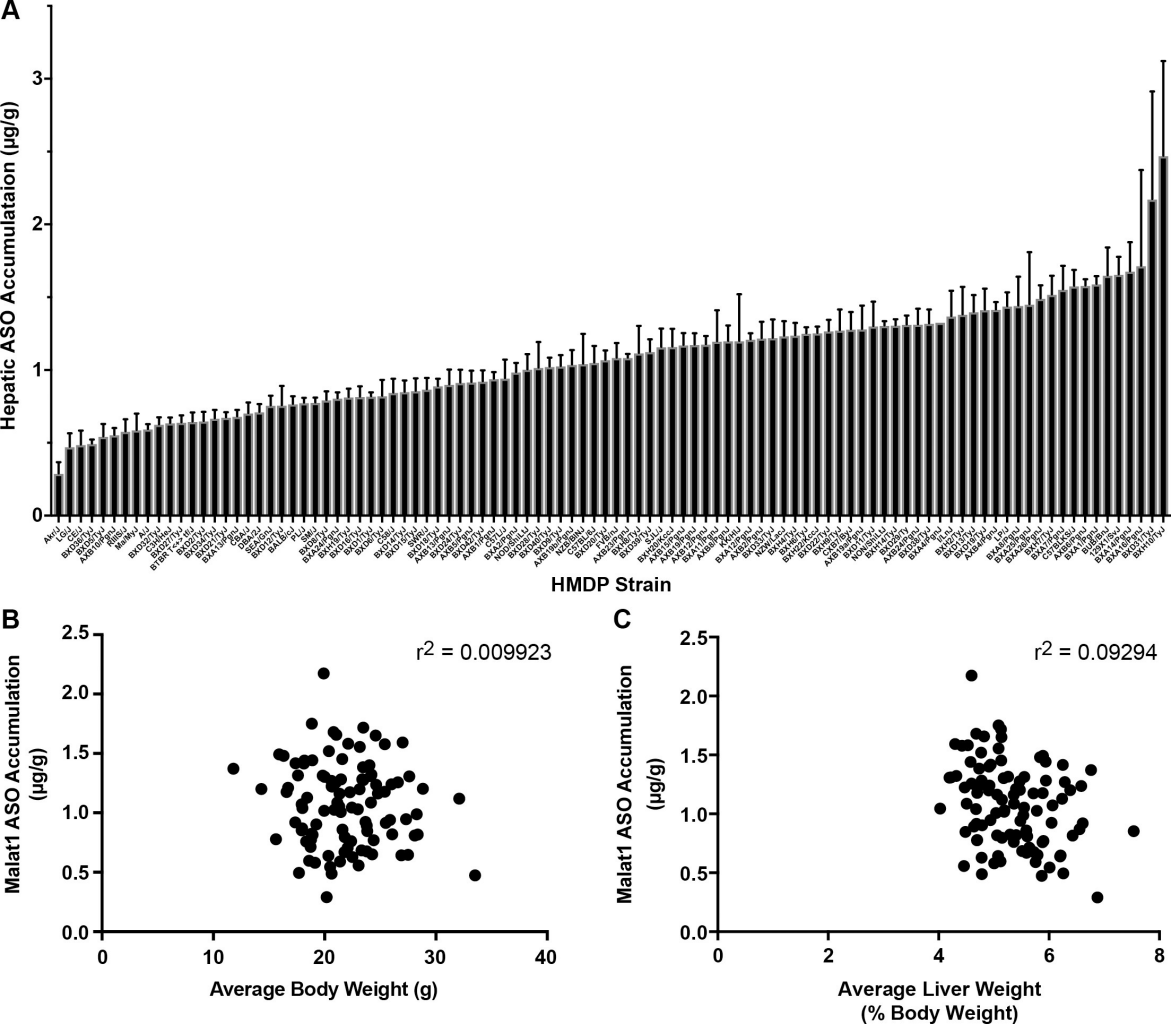
Effects of genetic background on hepatic accumulation of Malat1 ASO in the HMDP. (A) 6-week-old male mice from 100 HMDP strains (n = 5/strain/treatment) were dosed with a single 2 mg/kg dose of either Malat1 ASO (ION 556089) or control ASO (ION 549144). Livers were harvested after 72 hours and PK analysis performed as described. Results are presented as mean ± S.E.M. (B) Correlation of ASO PK with average body weight (BW) in grams and (C) average liver weight (expressed as % BW).

To identify genomic regions associated with Malat1 ASO PK, 6-week-old male mice from the HMDP were administered a single 2 mg/kg dose of either the constrained ethyl (cEt) Malat1 ASO (ION 556089) or the cEt control ASO (ION 549144), which does not target any known coding gene in the mouse genome (S1 Fig). A wide spectrum in hepatic accumulation of the Malat1 ASO (Fig 1A) was observed, ranging from 0.29 μg/g (Akr/J mice) to 2.17 μg/g (BXD31/TyJ mice). This variability did not correlate to average body (Fig 1B) or liver weights (Fig 1C).

**Fig 2:**
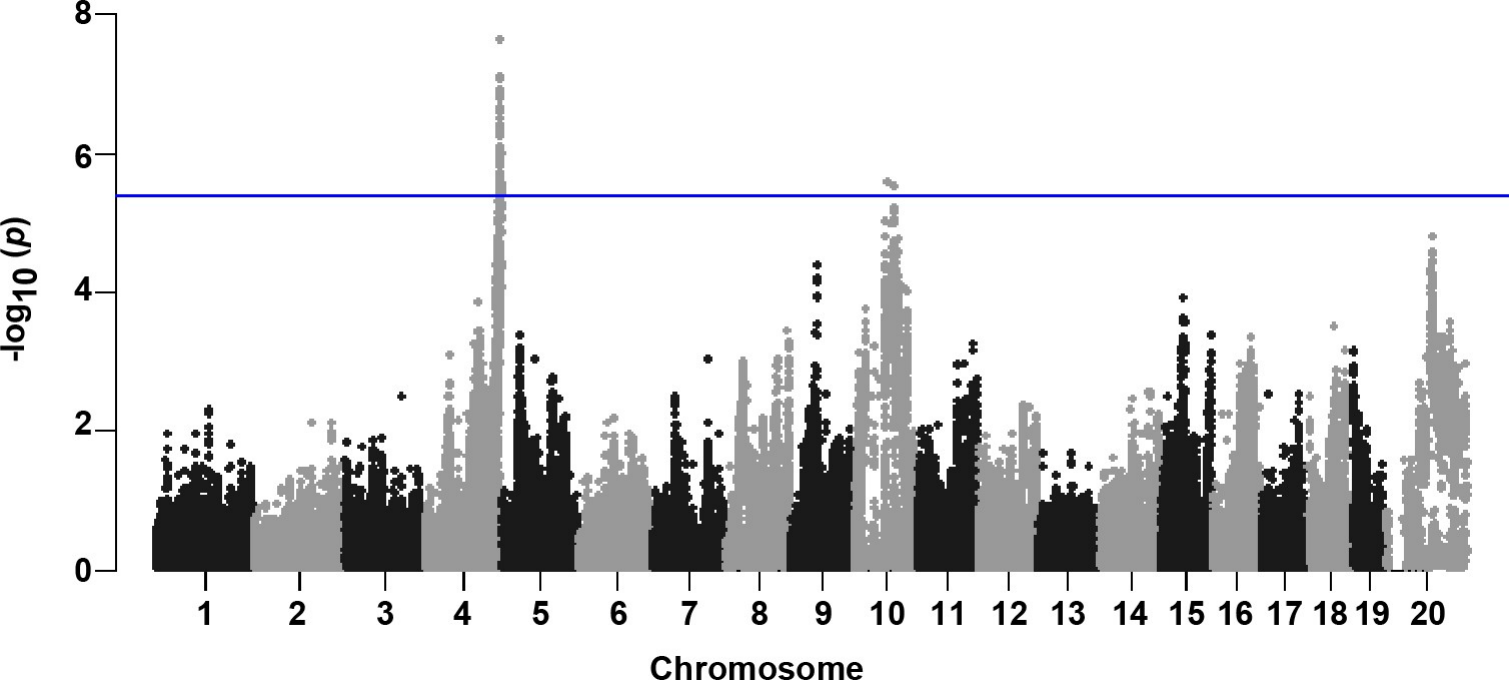
GWA results for hepatic Malat1 ASO concentrations in the HMDP. Manhattan plot showing the–log10 of the association p-values (-logp) for hepatic accumulation of Malat1 ASO in 100 HMDP strains. Each chromosome is plotted on the x-axis in alternating light and dark colors. Genome-wide significance threshold line is shown in blue (-logp = 5.39).

Association analysis was performed using FASTLmm in which adjusted association p-values were calculated for 108,064 SNPs with minor allele frequency of > 5% (p < 0.05 genome-wide equivalent for GWA using FASTLmm in the HMDP is p = 4.1 x 10^−6^, -log_10_p = 5.39). At this threshold, genome-wide significant loci associated with variation in hepatic Malat1 ASO accumulation (Fig 2) were identified on chromosomes 4 and 10 (rs32062485, p = 8.56x10^-10^; rs29346676, p = 1.93x10^-6^) (Table 1). The chromosome 4 locus contained 7 genes within the linkage disequilibrium (LD) block, out of which 5 were expressed in liver (Table 2). The chromosome 10 locus rs29364476 (Table 3) contained 11 genes, 6 of which were expressed in liver. These genes included *Stab2*, a type I transmembrane hyaluronan receptor involved in multiple cellular processes that has been previously implicated in affecting hepatic accumulation of phosphorothioate ASOs [28, 29].

**Table 1:** List of Peak SNP results for hepatic Malat1 ASO accumulation in the HMDP.

Trait	Sex	Chr.	Peak SNP	Position	p Value	LD	No. of Hepatic Genes
Hepatic MALAT-1 accumulation	male	4	rs32062485	153939301	8.56E-10	153.9–154.4	5
Hepatic MALAT-1 accumulation	male	10	rs29364476	87351304	1.90E-06	867.3–882.1	6

**Table 2:** List of genes within the linkage disequilibrium block for identified SNP rs32062485 in chromosome 4.

Gene Symbol	Gene Name	Expression
A430005L14Rik	RIKEN cDNA A430005L14 gene	Hepatic
Dffb	DNA Fragmentation Factor, beta subunit	Hepatic
Cep104	Centrosomal Protein 104	Hepatic
Lrrc47	Leucine rich repeat containing 47	Hepatic
Smim1	Small Integral Membrane Protien 1	Hepatic
BC039966	cDNA sequence BC039966	Non-Hepatic
Ccdc27	Coiled-coil domain containing 27	Non-Hepatic

**Table 3:** List of genes within the linkage disequilibrium block for identified SNP rs29364476 in chromosome 10.

Gene Symbol	Gene Name	Expression
Hsp90b1 (Rik)	Heat shock protein 90, beta	Hepatic
Stab2	Stabilin 2	Hepatic
Igf1	Insulin-like growth factor 1	Hepatic
Pah	Phenylalanine hydroxylase	Hepatic
Nup37	Nudeoporin 37	Hepatic
Ccdc53	Coiled-coil domain containing 53	Hepatic
Nt5dc3	5'-nucleotidase domain containing 3	Non-Hepatic
Ascl1	Achaete-scute complex homolog 1	Non-Hepatic
Pmch	Pro-melanin-concentrating hormone	Non-Hepatic
Parpbp	PARP1 binding protein	Non-Hepatic
Dram1	DNA-damage regulated autophagy modulator 1	Non-Hepatic

### Systems genetics analysis of additional PK associated SNPs

Since the majority of SNPs identified in the HMDP occur in noncoding regions, it became imperative to not only identify genes within the same LD block, but also those with expression regulated by the identified SNPs. To this end, global gene expression microarrays conducted using the liver tissue of chow-fed male mice from 95 HMDP strains [30] (NCBI GEO GSE16780) were used in conjunction with the SNP data to generate a list of expression quantitative trait loci (eQTL). The eQTL analysis led to the identification of *cis*- (within 1 Mb of the peak SNP) and *trans*- (greater than 1 Mb from the peak SNP) regulated genes corresponding to the SNPs at Chromosome 4 (S1 Table) and Chromosome 10 (S2 Table). Vesicle associated membrane protein 3 (*Vamp3*) was determined to be strongly regulated in *cis* by identified chromosome 4 SNP rs32062485 (p = 2.4 x 10^−28^, S1 Table and S3A Fig) and in *trans* by identified chromosome 10 SNP rs29346676 (p = 6.3 x 10^−8^, S2 Table).

### Validation of *Stab2*’s role in hepatic ASO accumulation

**Fig 3:**
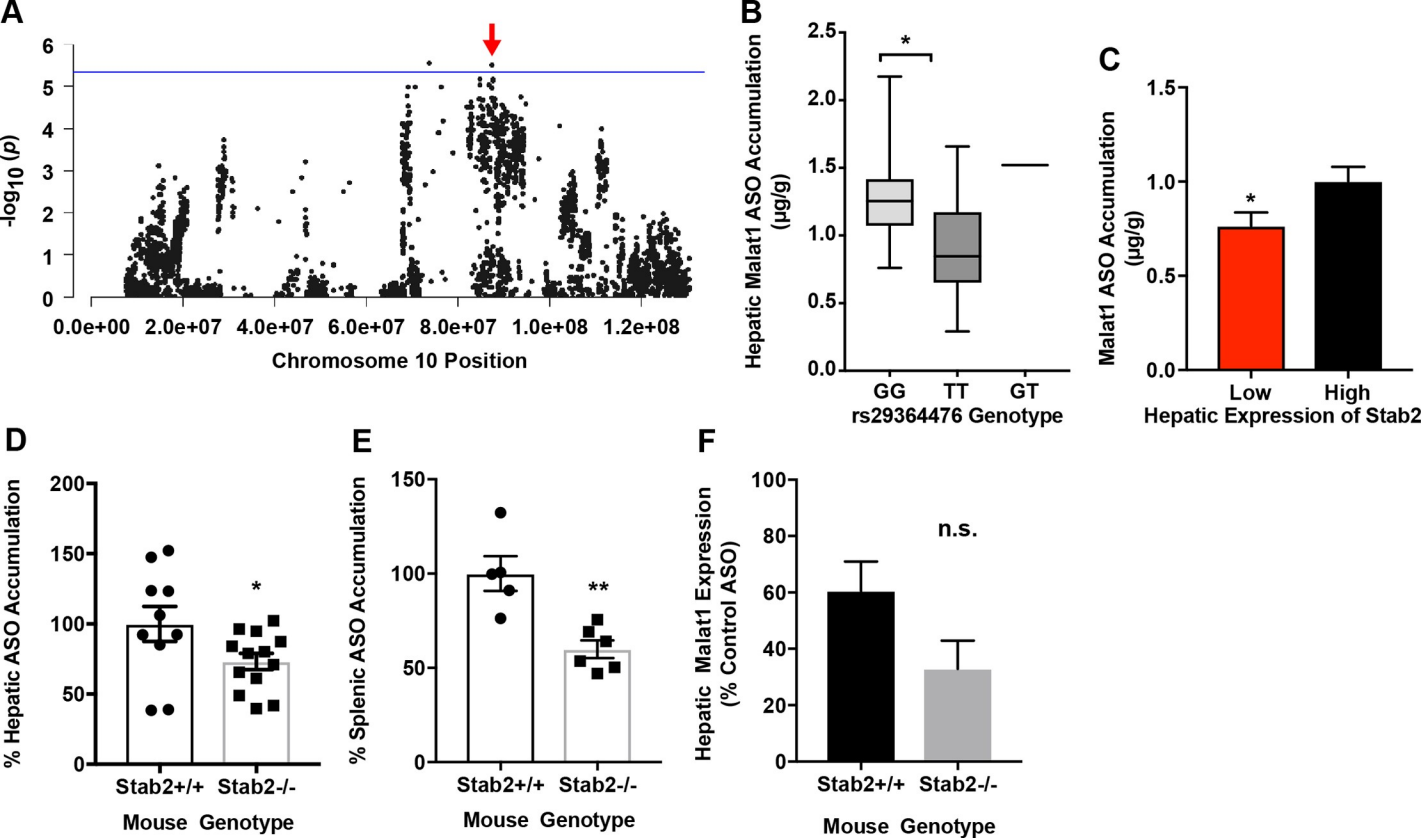
Systems genetics analysis and validation of *Stab2* for influencing hepatic accumulation of the Malat1 ASO. (A) Chromosome 10 plot for *Stab2* displaying all significant SNPs (-log10 of p) for ASO uptake on Chromosome 10. Red arrow indicates position of gene. (B) Distribution of Malat1 ASO accumulation based on genotype associated SNP on chromosome 10 (rs29364476). Box and whisker plot depicting mean and distribution. (C) HMDP strains with lower hepatic *Stab2* expression (S2 Fig) demonstrate lower hepatic ASO accumulation than those with higher *Stab2* expression. Mean ± SEM, * p ≤ 0.05 unpaired t-test, Welch’s correction. (D) Hepatic accumulation of Malat1 ASO was assessed in *Stab2*^-/-^ and WT mice 72 hours after single Malat1 ASO dose of 2 mg/kg. Hepatic concentration of ASO is significantly lower in livers of *Stab2*^-/-^ mice as compared to WT mice. Mean ± SEM, * p ≤ 0.05 unpaired t-test. (E) Splenic accumulation of Malat1 ASO was assessed in *Stab2*^*-/-*^ and WT mice 72 hours after single Malat1 ASO dose of 3mg/kg. Data shows mean ± S.E.M, * p≤ 0.05 ** p ≤ 0.01, unpaired t-test. (F) Expression of hepatic Malat1 mRNA by QPCR after treatment with Malat1 ASO compared to a control ASO. There is no significant difference between the *Stab2*^*+/+*^ and *Stab*^*-/-*^ mice, unpaired t-test. Data shows mean ± S.E.M.

*Stab2*, a previously determined key player in ASO accumulation [28, 29, 31], was identified within the LD block of peak chromosome 10 SNP rs29364476, and was further determined to be strongly regulated in *cis* by this SNP (p = 3.48x10^-6^, Fig 3A, S2 Table). Systems analysis of the peak SNP demonstrated a significant difference in hepatic ASO concentration dependent upon the genotype of SNP rs29364476 (Fig 3B). Based on the variation of hepatic expression of *Stab2* across the 100 strains, we next identified the strains expressing low and high levels of *Stab2* (Fig 3C, S2 Fig) and compared the hepatic drug tissue accumulation between the two groups via LCMS. Mice with lower *Stab2* expression accumulated significantly less drug compared to strains with higher *Stab2* expression (Fig 3C). To further confirm the role of *Stab2*, we utilized previously published *Stab2*^*-/-*^ mice [28]. Compared to WT (*Stab2*^*+/+*^ littermates), *Stab2*^*-/-*^ mice demonstrated significantly less ASO accumulation in both liver and spleen after a single Malat1 ASO dose (Fig 3D and 3E). Importantly, there was a trend but no significant difference in the ASO efficacy between the *Stab2*^*+/+*^ and *Stab2*^*-/-*^ mice (Fig 3F).

### Validation of *Vamp3’s* role in hepatic ASO accumulation

**Fig 4:**
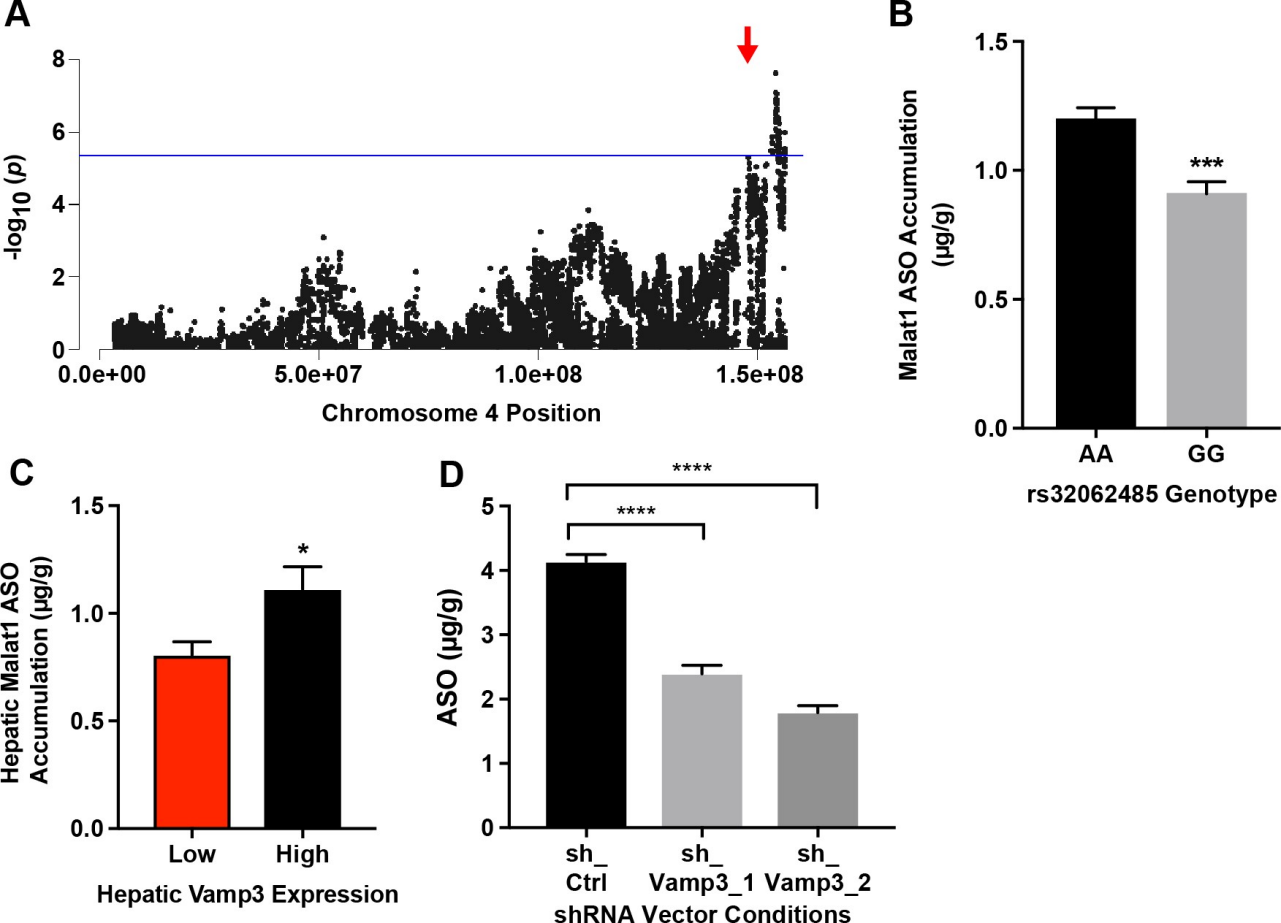
Systems genetics analysis and validation of *Vamp3* for influencing hepatic accumulation of the Malat1 ASO. (A) Chromosome 4 plot for *Vamp3* displaying all significant SNPs (-log10 of p) on Chromosome 4 for ASO uptake. Red arrow indicates position of gene. (B) Distribution of hepatic Malat1- ASO accumulation based on genotype distribution at peak SNP associated with ASO accumulation on chromosome 4 (rs32062485) data shows mean ± SEM, * p ≤ 0.05 unpaired t-test. (C) BXD strains with lower hepatic *Vamp3* expression (S3A Fig) displays significantly lower hepatic accumulation of Malat1 ASO. Data shows mean ± S.E.M, * p ≤ 0.05, unpaired t-test. (D) MHT cells transduced with shRNA targeting either scrambled control (shCtrl) or Vamp3 (sh_Vamp3_1, sh_Vamp3_2) were treated with 250 ug Malat1 ASO for 24 hours, then assessed for ASO uptake via LCMS. Cells with reduced Vamp3 expression had significantly lower ASO uptake. Data shows mean ± S.E.M, *** p< 0.0001 with one way ANOVA, Dunnett’s multiple compairisons test.

The *Vamp3* gene product is a small soluble N-ethylmaleimide-sensitive factor attachment protein receptors with Arg/R residue (R-SNARE) highly expressed in the liver and known to play a vital role in providing specificity in catalyzing the fusion of vesicles to their target membrane [32]. Since *Vamp3* plays a key role in vesicular transport, a process that has been implicated in ASO uptake, we investigated the putative role of *Vamp3* in hepatic ASO PK. As with *Stab2*, systems analysis of the peak SNP rs32062485 demonstrated a significant difference in hepatic ASO concentrations based on the genotype distribution at that SNP (Fig 4B). In order to isolate the effect of *Vamp3* expression and reduce the input of confounding genes, we utilized the BXD subset of the HMDP panel, which is derived from a single founder pair cross and shown previously to provide sufficient power in GWAS [23]. Our analyses determined that BXD strains with relatively lower *Vamp3* expression demonstrated significantly lower hepatic ASO concentrations (Fig 4C, S3A Fig).

In order to experimentally validate the role of *Vamp3* in ASO accumulation, we utilized a mouse hepatocellular SV40 large T-antigen carcinoma (MHT) cell line, which is capable of free ASO uptake. Cells were transduced with shRNA targeting either *Vamp3* or a scrambled control, then assessed for Vamp3 expression reduction by QPCR (Fig 4D). Cells were subsequently treated with 250 ug Malat1 ASO for 24 hours. LCMS of cells transduced with shRNA targeting Vamp3 demonstrated a significant decrease in ASO accumulation, validating Vamp3 as an important mediator of ASO uptake (Fig 4D).

### Genome-wide association analysis of hepatic ASO activity

**Fig 5:**
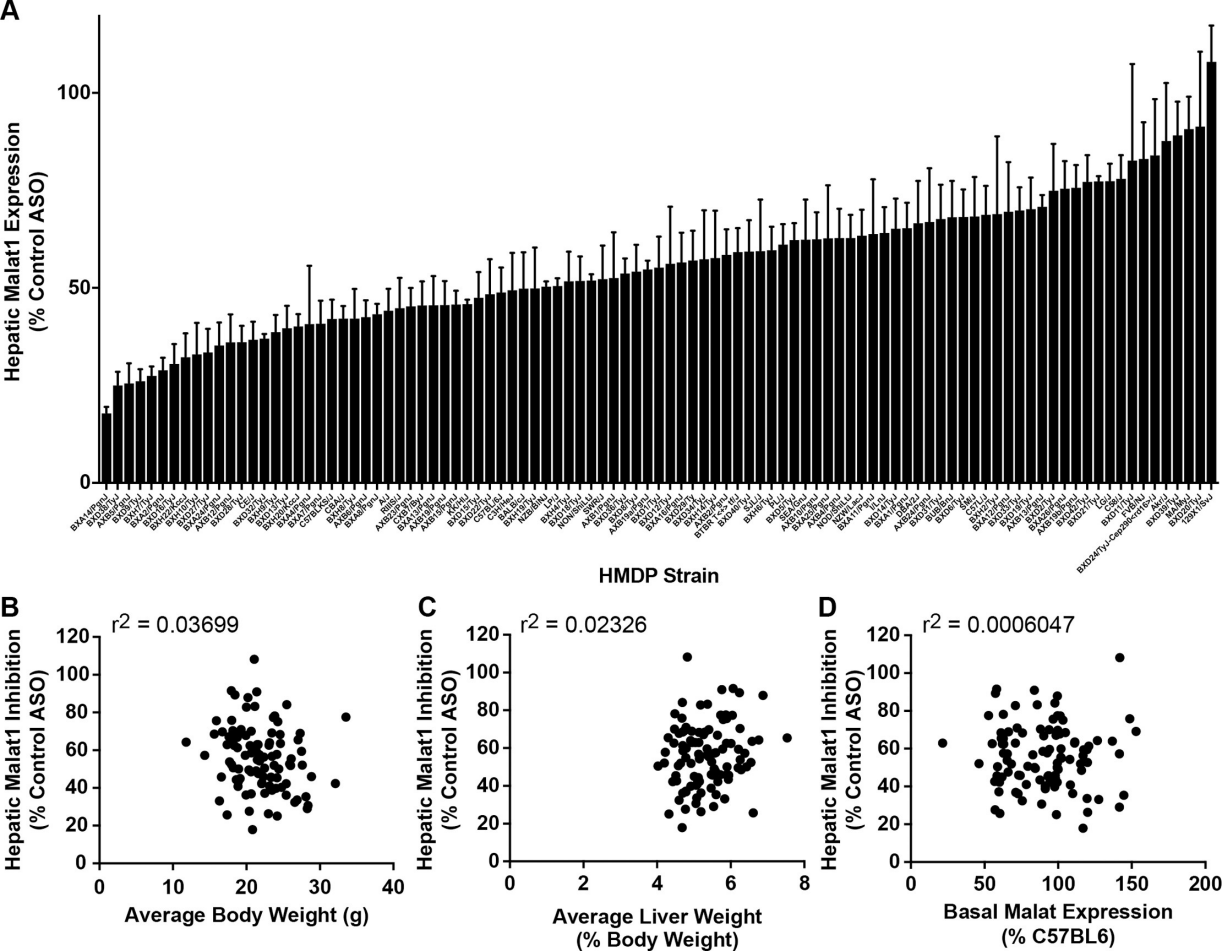
Large genetic variation in hepatic potency of the Malat1 ASO among male mice. (A) Hepatic *Malat1* expression in HMDP strains following a single dose of 2mg/kg of the Malat1 and control ASOs. Results are presented as mean ± S.E.M (% Control ASO). (B) Correlation of hepatic *Malat1* expression with average body weight (BW) in gm and (C) Average liver weight (expressed as % BW) (D) Correlation of hepatic *Malat1* knockdown and basal level of *Malat1* expression in liver of the 100 strains (as compared to C57BL/6J).

There was also marked variability in hepatic Malat1 ASO activity (Fig 5A), ranging from 82% target reduction in BXA14/PgnJ mice to essentially no activity (0%) in 129X1/SvJ mice. These results were confirmed via *in situ* immunostaining for Malat1 mRNA in ASO treated liver samples from the AXB5/PgnJ and BXD39/TyJ mice (S4 Fig). This genetic variability in ASO activity did not correlate with either average body or liver weights (Fig 5B and 5C) or hepatic Malat1 mRNA expression in control ASO treated mice (Fig 5D and S5 Fig). We also performed a dose response study and a time-course analysis of Malat1 ASO activity in three classic inbred strains of mice and results from both the studies were consistent with the single dose Malat1 experimental results (S4B and S4C Fig).

**Fig 6:**
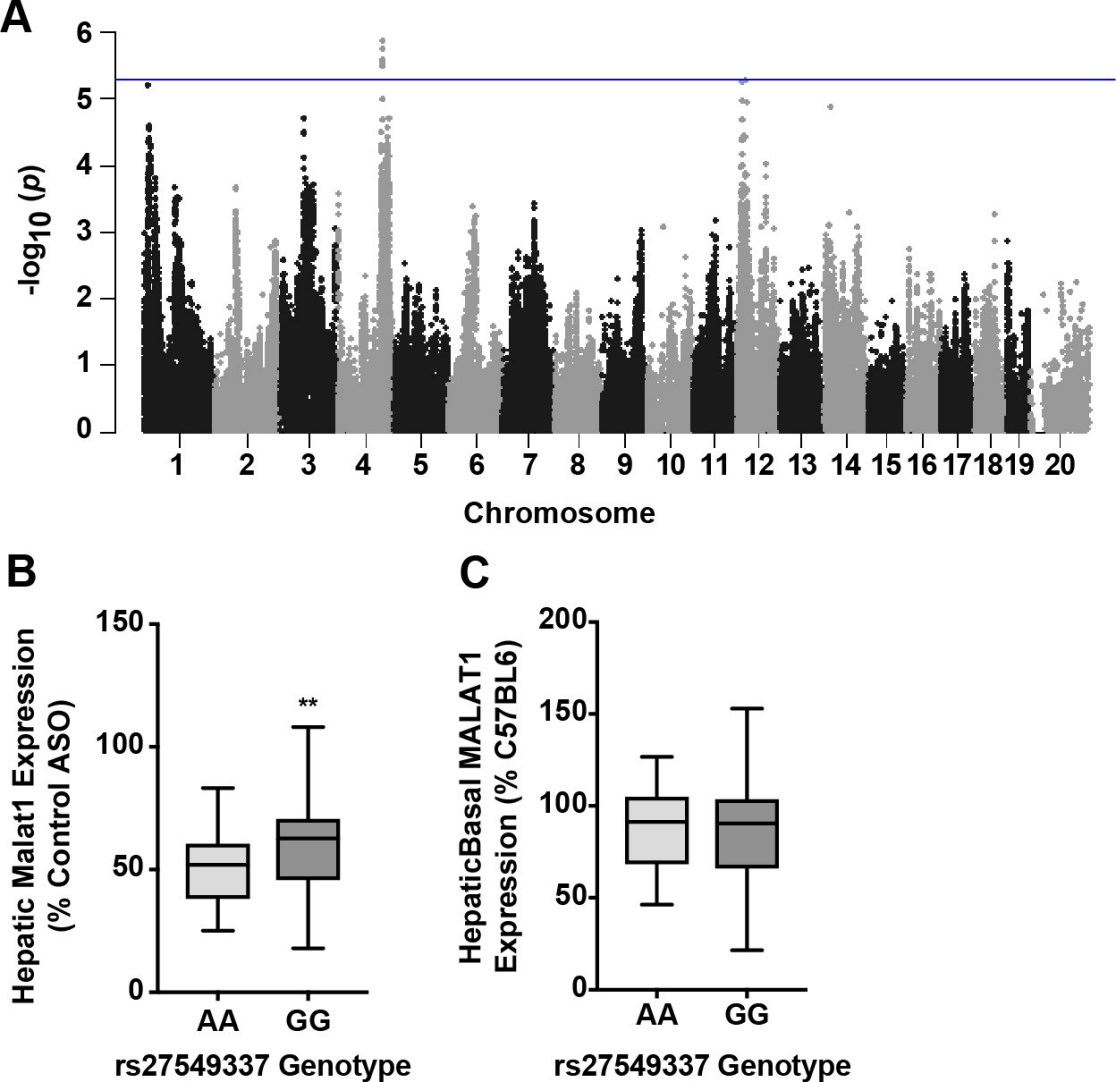
GWA results and validation for hepatic Malat1 ASO activity in the HMDP. (A) Manhattan plot showing the–log10 of the association p-values (-logp) for hepatic expression of *Malat1* mRNA in 100 HMDP strains. Each chromosome is plotted on the x-axis in alternating light and dark colors. Genome-wide significance threshold line is shown in blue (-logp = 5.39). (B) Distribution of hepatic *Malat1* expression based on genotype distribution at peak SNP rs27459337. Data shows box-and-whisker plot showing mean and distribution, unpaired t-test, ** p≤0.01. (C) Distribution of basal hepatic *Malat1* expression based on genotype distribution at rs27459337. Box-whisker-plot showing mean and distribution, unpaired t-test, n.s.

To identify genomic loci responsible for the variation in potency, we performed FASTLmm analysis with the genome-wide significance threshold of 4.1 x 10^−6^ and identified three loci (Fig 6A, Table 4): two loci on chromosome 12 (rs29210579 p = 5.4 x 10^−6^ and rs229212236 p = 5.5 x 10^−6^), which were 8.4 Mb apart. rs29210579 contained 9 genes with in the LD block, 7 of which are expressed in liver, while rs29212236 contained 6 genes within the LD block with 4 expressed in liver (Tables 5 and 6). The remaining locus was one on chromosome 4 (rs27549337, p = 1.33x10-6), with one hepatic gene in the LD block (Table 7).

**Table 4:** List of peak SNP results for hepatic Malat1 ASO activity in the HMDP.

Trait	Sex	Chr.	Peak SNP	Position	p Value	LD	No. of Hepatic Genes
Hepatic *Malat1* expression	male	12	rs29210579	16857727	5.40E-06	138.5–170.6	7
Hepatic *Malat1* expression	male	12	rs29212236	25280545	5.50E-06	252.8–235.1	4
Hepatic *Malat1* expression	male	4	rs27549337	126768234	1.33E-06	126.7–127.2	1

**Table 5:** List of genes within the linkage disequilibrium block for identified SNP rs29210579 in chromosome 12.

Gene Symbol	Gene Name	Expression
Psmb2	proteasome (prosome, macropain) subunit, beta type 2	Hepatic
AU040320	expressed sequence AU040320	Hepatic
Zmym4	zinc finger, MYM-type 4	Hepatic
Sfpq	splicing factor proline/glutamine rich (polypyrimidine tract binding protein associated)	Hepatic
Zmym1	zinc finger, MYM domain containing 1	Hepatic
Zmym6	zinc finger, MYM-type 6	Hepatic
Tmem35b	transmembrane protein 35B	Hepatic
Tfap2e	transcription factor AP-2, epsilon	Non-Hepatic
Ncdn	neurochondrin	Non-Hepatic

**Table 6:** List of genes within the linkage disequilibrium block for identified SNP rs2912236 in chromosome 12.

Gene Symbol	Gene Name	Expression
Lpin1	lipin 1	Hepatic
Rock2	Rho-associated coiled-coil containing protein kinase 2	Hepatic
E2f6	E2F transcription factor 6	Hepatic
Greb1	gene regulated by estrogen in breast cancer protein	Hepatic
Ntsr2	neurotensin receptor 2	Non-Hepatic
Pqlc3	PQ loop repeat containing	Non-Hepatic

**Table 7:** List of genes within the linkage disequilibrium block for identified SNP rs27549337 in chromosome 4.

Gene Symbol	Gene Name	Expression
Gm17746	predicted gene, 17746	Hepatic

### Systematic identification, systems genetics analysis and validation of genes associated with Malat1 ASO activity

Consistent with the accumulation studies described above, we again performed transcriptomic (eQTL) analyses using hepatic microarray expression data, accessible on NCBI GEO (GSE16780). This effort resulted in a list of genes that were in *cis* and *trans* regulation by the peak SNP at chromosome 4 (rs27549337) and two on chromosome 12 (rs29210579 and rs29212236) (S3, S4 and S5 Tables).

Analysis of SNP rs27549337 on chromosome 4 indicated that variability of the ASO activity associated with this SNP was not associated with endogenous Malat1 mRNA expression variability, further validating the systems genetics approach (Fig 5B and 5C). Four genes were revealed to be regulated by that SNP in *cis*. Three of these genes are expressed in liver, and intriguingly the list includes *Utp11l*, small subunit processome component [33]. This protein is ubiquitously expressed and is involved in active pre-RNA processing complex, making it an appealing target [34].

Systems analysis of the chromosome 12 SNP rs29210579, as with the chromosome 4 SNP, indicated that the variability in Malat1 ASO activity associated with the SNP was not due to basal *Malat1* mRNA expression level differences (Fig 6A and 6B). We identified *Rock2* and *Lpin1* as high-confidence candidate genes because they both were (i) expressed in the liver, (ii) present in the LD region (Table 6) and, (iii) strongly regulated in *cis* by rs29210579 (p = 1.27x10^-10^ S4 Table).

To assess the function of *Lpin1* in modulating ASO potency, we again utilized a mouse hepatocellular SV40 large T-antigen carcinoma (MHT) cell line [35], with non-silencing (NS) siRNA or *Lpin1* siRNA for 48 h. Treatment with *Lpin1* siRNA yielded a 58% reduction in target gene expression (S6A and S6B Fig). Subsequent exposure of siRNA treated cells to Malat1 ASO for 72 hours did not reveal differential ASO activity (S6C and S6D Fig) suggesting that *Lpin1* is not playing a significant role in Malat1 ASO activity under these conditions.

### Validation of *Rock2’s* role in hepatic ASO activity

**Fig 7:**
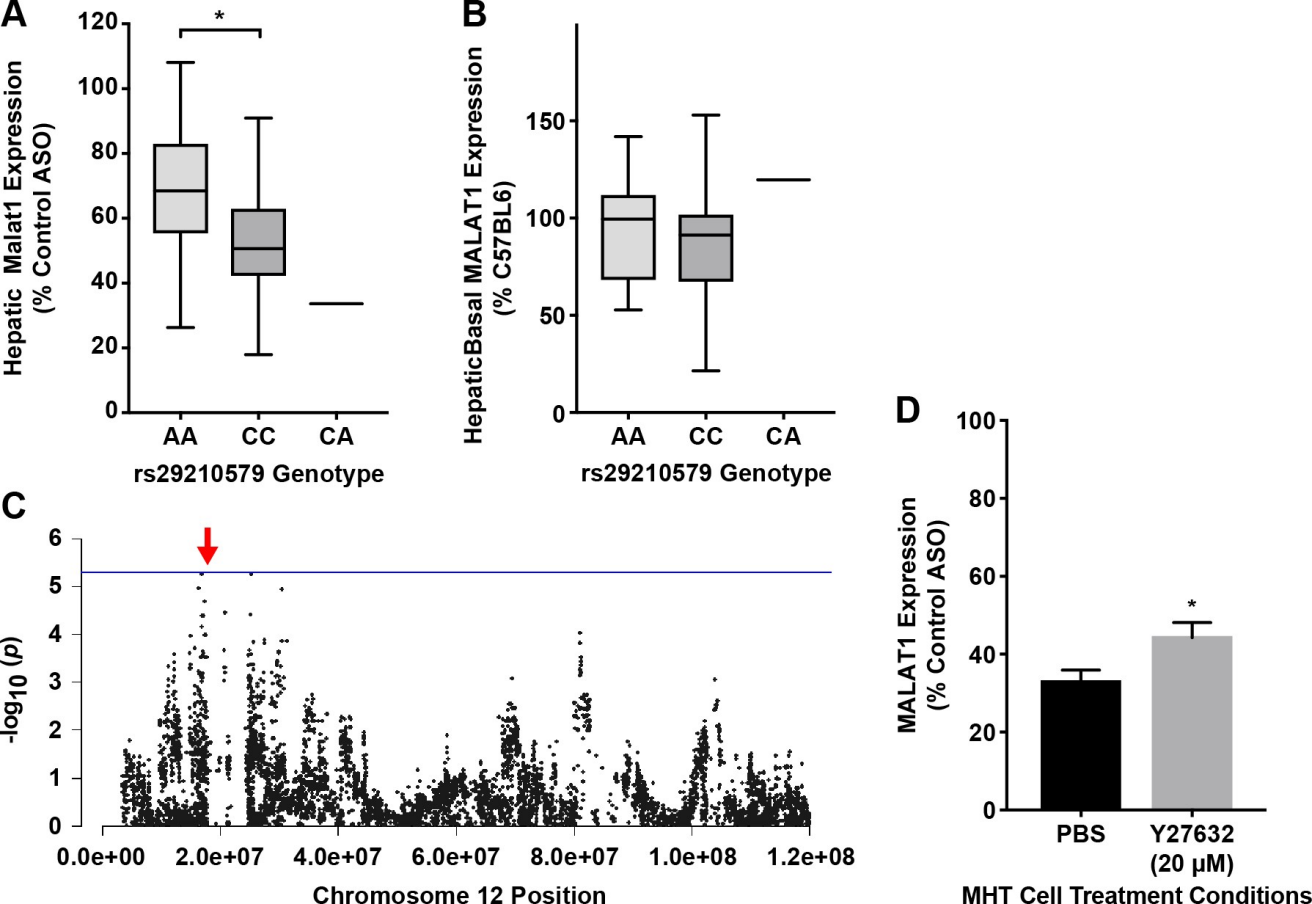
Identification and validation of Rock2 in ASO activity. (A) Distribution of hepatic *Malat1* expression based on genotype distribution at rs29210579. Box and whisker plot depicting mean and distribution, unpaired t-test, * p≤0.05 (B) Distribution of basal hepatic *Malat1* expression based on genotype distribution at rs29210579. Box and whisker plot depicting mean and distribution. (C) Chromosome 12 plot for *Rock2* displaying all significant SNPs (-log10 of p) for ASO efficacy. Red arrow indicates position of gene. (D) MHT cells were incubated with Malat1 ASO and control ASO in presence and absence of 20 μM Y27632. After 24 hrs, Malat1 ASO potency was assessed in all samples. MHT cells exhibit lower reduction in *Malat1* expression with Y27632 as compared to PBS. Data shows mean ± S.E.M (% control ASO) with two-way ANOVA and Bonferroni’s multiple comparison test. * p≤ 0.05.

To inhibit the kinase activity of *Rock*, MHT cells were treated for 24 h with Y27632, a potent pharmacological inhibitor of *Rock1* and *Rock2*. In the presence of Y27632 a significant decrease in phospho-myosin light chain 2 (*MLC2*) was observed (S6E Fig), consistent with inhibition of *Rock* kinase activity. Subsequent exposure of treated cells to Malat1 ASO revealed a significant difference in ASO activity in treated vs nontreated MHT cells (Fig 7D), validating our finding that *Rock2* regulation impacts ASO activity.

### Validation of *Adi1’s* role in hepatic ASO activity

**Fig 8:**
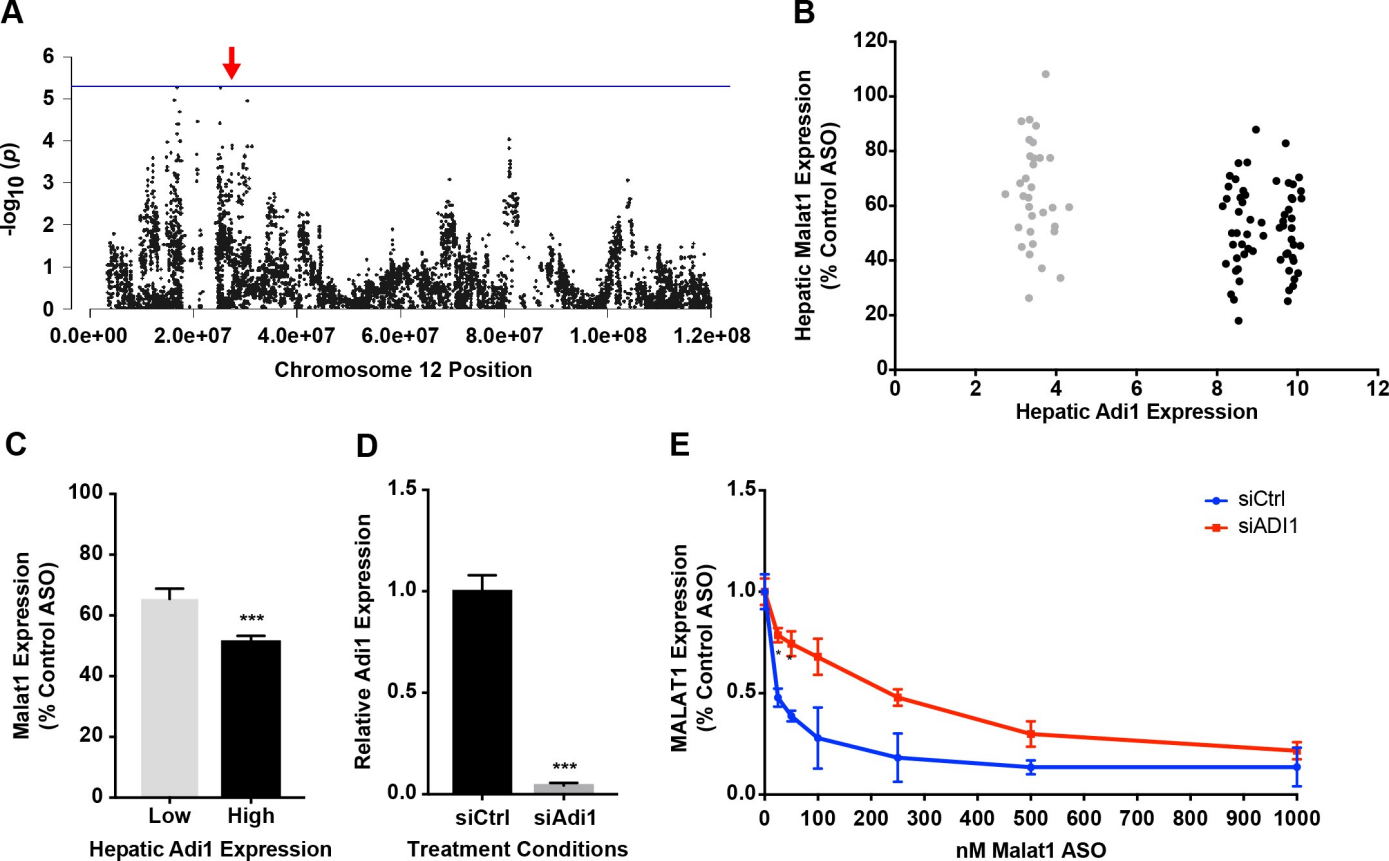
Identification and validation of *Adi1* in ASO activity. (A) Chromosome 8 plot for *Adi1* displaying all significant SNPs (-log10p) for ASO efficacy on chromosome 8. Red arrow indicates position of gene (B) Plot of hepatic *Adi1* expression and hepatic potency of 556089 shows differential *Adi1* expression among the HMDP strains. Low expressing strains are indicated in grey. (C) HMDP strains with lower hepatic *Adi1* expression show higher potency compared to strains with higher hepatic *Adi1* expression. **** p ≤ 0.0001, unpaired t-test (D) *Adi1* expression in MHT cells treated with either control siRNA or Adi1 siRNA. Data shows mean mean ± S.E.M *** p < 0.001, unpaired t-test (E) Plot of Malat ASO efficacy across indicated doses in MHT cells treated with Control siRNA or Adi1 siRNA. Data shows significant differences in ASO activity at 25 and 50nM Malat1 ASO, multiple t-tests using the Holm-Sidak method, with alpha = 0.05. * p ≤ 0.05.

We also considered other *cis-*genes that may affect ASO PD. Aci-reductone dioxygenase (*Adi1*) was the gene most significantly regulated (p = 3.4x10^-38^, S5 Table and Fig 7A) in *cis* by chromosome 12 SNP rs29212236. While *Adi1* is primarily known for its role in methionine metabolism, the human orthologue has recently been implicated to a novel role in nuclear mRNA processing possibly by modulating splicing factor U1-70K-related functions [36]. Systems analysis of *Adi1* reveals that strains with higher *Adi1* had lower Malat1 ASO activity (Fig 8B and 8C), thus suggesting that interactions between several genetic factors might affect the potency of ASO drugs.

In order to futher validate the role of *Adi1* in ASO activity, MHT cells were treated with either a scrambled siRNA or *Adi1* siRNA for 24 hrs, washed, and subsequently exposed to increasing concentrations of Malat1 ASO (Fig 8D). Treatement with *Adi1* siRNA showed a significant reduction in ASO activity compared to control siRNA, validating *Adi1* as an important mediator of ASO efficacy in liver (Fig 7E).

## Discussion

Antisense drugs have been successfully used as research tools and as therapeutic agents in the clinic for decades. While the basic uptake and activity properties of these drugs have been elucidated [37], ongoing research efforts seek to identify factors that affect the distribution, efficacy, safety and tolerability of these therapeutic agents [38]. Advances in ASO medicinal chemistry have drastically enhanced activity, allowing significantly lower doses of drug to be administered in the clinic. Recently, research has been increasingly directed toward understanding the mechanisms of oligonucleotide uptake [39]. Briefly, once ASOs reach the cell surface they are internalized via endosomal vesicles, ultimately reaching their intended mRNA target either in the nucleus or cytosol [14, 39, 40]. It is generally accepted that some pathways of internalization and trafficking are productive, i.e leading to a pharmacological effect, while others are pharmacologically non-productive ‘sinks’ [41, 42]. Several structural, nucleic acid binding and chaperone proteins have been shown to bind to ASOs and influence intracellular localization and trafficking within the cell [42, 43]. Most importantly, some of these interactions are known to impact ASO therapeutic potency [4].

Here we investigate the role of genetics in modulating hepatic Malat1 ASO uptake and activity in mice. Results from our studies demonstrate that after a single dose of a Malat1 cEt-modified ASO, hepatic distribution of the drug and activity vary significantly among the 100 genetically unique HMDP strains. This considerable variability in both parameters allowed us to perform GWAS and led to the identification of two genes, *Stab2* and *Vamp3*, associated with variation in hepatic ASO deposition, and another two, *Rock2* and *Adi1*, that are associated with variation in ASO potency. These results are particularly noteworthy as those four genes are involved in either internalization and endosomal/lysosomal trafficking processes known to be crucial for ASO uptake and subcellular localization [39, 44, 45]

Previous work has demonstrated *Stab2* is capable of binding and internalizing PS MOE ASOs in stabilin-expressing stable cell lines [28]. Additionally, immunostaining in wildtype mice displayed high ASO accumulation in tissues that expressed Stab2 [28, 46]. Here we expand on these previous findings and confirm the role of *Stab2* in hepatic ASO uptake. First, GWAS and systems analysis determined the *Stab2* gene located within a peak SNP LD block, as well as locally regulated by the presence of strong *cis-*eQTLs associated with the SNP haplotype. Second, HMDP strains with lower *Stab2* expression presented with significantly lower hepatic ASO accumulation when compared to strains with more highly expressed *Stab2*. Third, ASO immunohistochemistry has previously demonstrated that liver sinusoidal endothelial cells (LSECs), a site of high hepatic *Stab2* expression, exhibited relatively high concentrations of ASO [47]. Finally, *Stab2*^-/-^ mice accumulated significantly less hepatic and splenic Malat1 ASO when compared to WT mice. Significantly, the differences in Malat1 ASO uptake between *Stab2*^*-/-*^ and WT were more pronounced in spleen, which has higher *Stab2* expression [28]. While this evidence demonstrates that *Stab2* expression is critical in a subset of cells for ASO uptake, that both the livers and spleens of *Stab2*^-/-^ mice were capable of some ASO accumulation and the existence of cell types capable of ASO uptake which don’t express *Stab2* is evidence indicating the existence of *Stab2*-independent ASO uptake pathway(s) [48]. Surprisingly, when we assessed Malat1 antisense drug potency in the WT and *Stab2*^-/-^ mice, we did not see any significant changes in hepatic Malat1 ASO potency. In fact, a recent study has demonstrated that Scavenger Receptor B1 (*Srb1*) ASO had reduced potency in *Stab2*^*-/-*^ when compared to wildtype mice [41, 35]. A likely explanation for this discrepancy is the fact that, relative to *Malat1*, a larger proportion of hepatic *Srb1* mRNA expression is in LSEC cells versus hepatocytes [49, 39]. Thus, ablation of *Stab2* dependent uptake into LSECs would be expected to have more deleterious effect on SRB1 ASO potency relative to the Malat1 ASO that targets a more hepatocyte localized mRNA.

While the current work does not address the mechanism for the disconnect between hepatic Malat1 ASO accumulation and activity (S2 Fig), it is tempting to speculate that the *Stab2*-mediated ASO uptake/internalization pathway is predominantly higher capacity and lower affinity than other productive pathways [31, 41]. This is further validated by our data demonstrating that *Stab2* is implicated in ASO uptake but not efficacy (Fig 3). These data are consistent with evidence that there are numerous uptake/internalization pathways that can be broadly categorized as productive and non-productive with regards to antisense pharmacology [14]. To better understand the relationship between ASO accumulation and activity, we compared ASO liver accumulation and potency in the 100 strains and found that there was no correlation (S7 Fig). This lack of hepatic accumulation-activity correlation further supports the existence of multiple ASO uptake pathways, and that these pathways have varying levels of efficacy in delivering the ASO to its target RNA.

Rho-kinase (*Rock2*) is one of the serine/threonine kinases functioning as a downstream effector molecule of small GTPase RhoA promoting contractile force generation and morphological changes in cells and organs. *Rock2* has been shown previously to promote acto-myosin contractility and tubulin polymerization by either directly phosphorylating the myosin regulatory light chain (MLC) and the myosin binding subunit (*MYPT1*) of the MLC phosphatase or phosphorylating LIM kinases-1 and –2 (*LIMK1* and *LIMK2*) [50–53]. *Rock2* activity is known to play a role in the movement of endosomal and lysosomal vesicles [54, 55]. One gene that has been shown to be significant in the productive endosomal trafficking of ASO is *Anxa2*, a phospholipid binding protein that is required for the biogenesis of late endosomes [56]. Studies have shown that *Anxa2*is able to bind ASOs, and upon incubation of ASOs with cells ANXA2 was enriched in late endosomes, suggesting that *Anxa2*is important for intracellular conveyance of ASOs [42]. Interestingly, *Rock* activity is essential for several ANAX2 signaling pathways [57, 58]. Even though the interactions of *Anxa1* and *Rock2* on ASO activity remain unclear, it suggests that factors affecting endosomal trafficking are important for productive ASO uptake.

In summary, genetic factors have been shown for the first time to be capable of altering the hepatic ASO accumulation and activity of Malat1 cET ASO *in vivo*. Here we establish the utility of HMDP in identifying genes affecting the uptake and potency properties of ASOs; future work must focus on translating these findings to human systems. Future studies will include using *in vitro* and *in vivo* model systems to understand the specific mechanism(s) of action of these identified genes in ASO uptake and/or intracellular trafficking. Finally, further studies evaluating the factors affecting potency and uptake in disease models will provide us with a more concise understanding of ASO response spectrum across populations and will help us design more effective drugs with improved therapeutic benefits.

## Materials and methods

### Ethics statement

Ionis is AAALAC accredited and follows the 8th Ed. Of the Guide for the Care and Use of Laboratory Animals and the 2013 AVMA guidelines for the euthanasia of animals. All animals in this study were anesthetized with Isoflurane and euthanized via cervical dislocation. The Ionis IACUC-approved protocol is # P-0225. This protocol was approved on 5/28/2014.

### Oligonucleotide synthesis and delivery

2´, 4´- constrained 2´-O-ethyl (cEt) ASOs were synthesized at Ionis Pharmaceuticals (Carlsbad, CA) as described previously [59]. The Malat1 (ION 556089) and control (ION 549144) ASOs were formulated in saline, and injected via subcutaneous administration into the animals. In order to monitor reproducibility of the subcutaneous dosing, we used C57BL/6J mice as the control strain for each experiment. Similar levels of *Malat1* inhibition in the livers were observed for each experiment (S8 Fig).

### Mice

Mice were obtained from The Jackson Laboratory and housed at Ionis Pharmaceuticals (Carlsbad, CA), maintained on a chow diet and entered into studies before they exceeded 7 weeks of age. Stabilin-2 knockout (*Stab*2^-/-^) mice, developed as described in Hirose et.al. 2012 [60], were purchased from Riken BioResource Center. At the end of each experiment, mice were anesthetized, euthanized by cervical dislocation and blood was collected by cardiac puncture. Spleens and livers were harvested and immediately snap frozen in liquid nitrogen for mRNA expression analysis and histology. As many antisense drugs target liver [37, 61], our main analytical endpoints were hepatic drug accumulation and reduction of Malat 1 liver mRNA expression. Despite the hepatic abundance of *Malat1*, its loss of function has been found to be phenotypically silent, with no effect on global gene expression, phosphorylation, splicing factor levels or pre-mRNA splicing events [46, 62]. A dose response study of Malat1 ASO performed in 3 classic inbred strains (S9 Fig) determined the ED_50_ to be approximately 2 mg/kg. ASO tolerability and hepatotoxicity were assessed by measurement of serum aminotransferase (ALT and AST) and spleen weights (S10A, S10B and S10C Fig). Although significant baseline variability was observed in the serum ALT/AST levels and spleen weights, no post-dose elevations in either parameters were observed in any strains.

### Cell lines and reagents

MHT cells were cultured in DMEM supplemented with 10% fetal bovine serum, streptomycin (0.1 mg/ml), and penicillin (100 U/ml) as previously described [35]. Malat1 and control ASOs, and the ROCK inhibitor Y27632 (Tocris, Cat. No. 1254) were added to cells at the indicated concentrations for 24 hours. 5 nmole of Silencer select siRNA (Ambion, Negative Control #1, Lpin1 –s66131, ADI1 –s75892 and 186988, VAMP3 –s98387 and s98386) was formulated in RNAiMAX plus Opti-MEM (ThermoFisher Scientific) and incubated with cells for 48 hrs. shRNA targeting *Vamp3* was purchased from Origene (Cat. Nos. scrambled control: TR30021V, Vamp3_1: TL515358VC, Vamp3_2: TL515358VD). MHT cells cultured as described to a 50% confluency were exposed to 20uL viral particles for 24hrs. Cells were washed and selected with 1ug/mL Puromycin (Sigma Aldritch, Cat. No. P8833) for 10 days, then colonies were selected and plated in a 96 well plate. Target knockdown was assessed by QPCR and normalized to cyclophilin.

### RNA analysis

Total mRNA was isolated using a QIAGEN RNAeasy kit (QIAGEN, Valencia, CA, USA). Reduction of target mRNA expression was determined by qPCR using StepOne RT–PCR machines (Applied Biosystems) as described previously [35]. Relative levels of *Malat1* and *Lpin1* were normalized to Cyclophilin.

### Liver pharmacokinetic analysis

Pieces of whole liver were minced, weighed into individual wells, then homogenization buffer [63] was added to those corresponding wells. Control tissue homogenate was made by adding homogenization buffer at a 9 to 1 ratio to weighed amount of mouse untreated liver. Aliquots and appropriate amounts of calibration standards were added into wells. Internal standard and approximately 0.25cm^3^ granite beads were added and then extracted via a liquid-liquid extraction with ammonium hydroxide and phenol: chloroform: isoamyl alcohol (25:24:1). The aqueous layer was then further processed via solid phase extraction utilizing a Phenyl plate. Eluates had a final pass through a protein precipitation plate before dry down under nitrogen at 50°C. Dried samples were reconstituted in water containing 100 μM EDTA. These samples were injected into an Agilent LCMS instrument consisting of a 1260 binary pump, a 1200 isocratic pump, a column oven, an auto sampler, and a 6130 single quadrupole mass spectrometer for analysis (Agilent, Wilmington, DE, USA).

### Association analysis

Since genetic association studies in inbred mice produce inflated false positive results owing to population structure and genetic relatedness, it is essential to use appropriate statistical tests. Efficient mixed model association (EMMA) utilizes linear mixed model with single dimensional optimization and phylogenetic control based kinship analysis to account for population stratification [26]. EMMA enabled correction for the spurious associations helps to cut down on the data processing time manifold. We identified genetic associations using the FastLMM, which is a reformulated mixed model analysis that performs linearly in run time and memory footprint for GWAS in very large data sets [27]. We retrieved the genotypes from UCLA systems genetics database along with chromosomal locations of linkage disequilibrium (LD) blocks. The average LD block size for the HMDP is 2Mb. The blocks were calculated here using Plink2’s implementation of the Haploview algorithm and appear to be smaller than average (S6 Table). The global gene expression microarrays which were generated from liver of chow fed male mice from 95 HMDP strains, were obtained from the UCLA systems genetics database and are available at NCBI GEO (GSE16780). These data were used to perform the *cis-*and *trans-* analysis as previously described [30]. FastLMM on ASO accumulation and activity properties was performed with a genome-wide significance threshold of 4.1 x 10^−6^ as described [21–25, 30].

### Statistics

Data are reported as means ± SEM. The statistical tests are mentioned in the figure legends. Statistical significance was considered when *p*<0.05. Unpaired Student's t-test or two-way ANOVA and Bonferroni’s multiple comparison test have been used to determine significance relative to control groups.

## Supporting information

S1 Fig Antisense oligonucleotides (ASO) used for this study. (A) Structural illustration of cET ASOs and PS-MOE ASO showing the organic modifications in the 2’ and 4’ carbon of the ribose moiety and (B) the full length sequence of each specific ASO used in the study.
(TIF)

S2 Fig Hepatic *Malat1* expression after Malat1 ASO dose in WT and *Stab2*^-/-^ mice. Plot of hepatic *Stab2* expression and hepatic accumulation of Malat1 ASO. Red dots indicate the strains with low hepatic *Stab2* expression. These outlier strains with low hepatic *Stab2* expression were identified.
(TIF)

S3 Fig *Vamp3* expression regulates hepatic PK of Malat1 ASO. (A) Plot of hepatic *Vamp3* expression and hepatic accumulation of Malat1 ASO in BXD strains. Grey dots indicate the strains with low hepatic *Vamp3* expression (B) Relative *Vamp3* mRNA expression in MHT cells after transduction with either scrambled control or *Vamp3* targeting shRNA. Data shows mean ± S.E.M * p ≤ 0.05.
(TIF)

S4 Fig Verification of hepatic *Malat1* expression. (A) Liver tissue from AXB5/PgnJ and BXD39/TyJ showing *Malat1* expression (*Malat1* staining is shown in red). AXB5/PgnJ shows a higher reduction *Malat1*expression than BXD39/ TyJ which is commensurate with hepatic potency of Malat1 ASO in the two strains. Scale = 50 um (B) 72 hour SD dose-response curve in three classic inbred HMDP strains with 0.2, 0.7, 1.4, 2, 4, 6 and 8 mg/kg of Malat1 ASO. (C) Hepatic *Malat1* expression in three classic inbred HMDP strains with different time points of incubation (6 hrs, 24 hrs, 48 hrs and 72 hrs) with Malat1 ASO. Data points are mean ± S.E.M.
(TIF)

S5 Fig Relative hepatic *Malat1* expression in 100 HMDP strains as compared to expression in C57BL/6J strain. Significant variability was observed in basal hepatic *Malat1* expression levels among the 100 strains of HMDP as seen with single 2mg/kg Malat1 ASO dose after 72 hrs. Data points are mean ± S.E.M.
(TIF)

S6 Fig Validation of *Lpin1* and *Rock2* inhibition after treatment with siRNAs and Y27632 and Malat1 ASO dose response curve in MHT-1 cells. (A) Inhibition of *Lpin1* in MHT-1 cells after 48 hours incubation with 32 nM siRNA (B) Western blot analysis to confirm knockdown of Lipin1 (normalized to GAPDH) (C) MHT-1 cells were incubated with 25, 50, 100, 250, 500 and 1000 nM Malat1 ASO for 72 hours. The IC_50_ was calculated to be 216.5 nM. (D) No significant change in Malat1 ASO potency observed with inhibition of Lpin1 with Lpin1 siRNA as compared to NS siRNA. (E) Western blot analysis confirms downregulation of ROCK activity with significant decrease in pMLC2 levels. Quantification was normalized to MLC2. Data represents mean ± S.E.M. * p ≤ 0.05 ** p ≤ 0.01, *** p ≤ 0.001, **** p ≤ 0.0001, unpaired t-test.
(TIF)

S7 Fig Correlation between Malat1 ASO hepatic potency and accumulation. Hepatic potency and accumulation of Malat1 ASO, obtained post single dose of 2mg/kg ASO, in the 100 HMDP strains were correlated. No correlation was observed between hepatic PK and PD of Malat1 ASO for 100 strains.
(TIF)

S8 Fig Hepatic *Malat1* expression in C57BL/6J. C57BL/6J animals were used in every experiment as a control strain to ensure reproducibility of data. 6 week old male C57/BL/6 animals (n = 4/ strain/treatment) were administered a single dose of 2 mg/kg of ION 556089 (black bar) or ION 549144 (grey bar). After 72 hours, gene expression was assessed in the harvested livers. Similar trends in reduction of hepatic *Malat1* expression (normalized to control ASO) were obtained in most of the experiments. Data points are mean ± S.E.M.
(TIF)

S9 Fig Effective dose 50 (ED_50_) determination of Malat1 cET ASO in HMDP mice strains. 6 week male mice from Balb/cJ, C57BL6/J and 129x1/SvJ were (n = 5/strain/treatment) were dosed subcutaneously with 1, 3, 5 and 10 mg/kg of either Control cET ASO (ION 549144) or Malat1 cET ASO (ION 556089). Hepatic *Malat1* mRNA expression was assessed 72 hours post injection using qRTPCR and results are presented as mean ± S.E.M.
(TIF)

S10 Fig Serum aminotransferase and spleen weights after ASO administration in HMDP strains. (A) Alanine transaminase (ALT) and Aspartate transaminase (B) levels in serum and (C) spleen weights in the 100 inbred strains after 2mg/kg single dose of Malat1 ASO as compared to Control ASO, 72 hrs post-injection. Data points are mean ± S.E.M.
(TIF)

S1 Table *cis* and *trans* eQTLs associated with Chromosome 4 SNP rs32062485, p = 4.1x10^-6^. (PDF)

S2 Table *cis* and *trans* eQTLs associated with Chromosome 10 SNP rs29364476, p = 4.1x10^-6^. (PDF)

S3 Table *cis* and *trans* eQTLs associated with Chromosome 4 SNP rs27549337, p = 4.1x10^-6^. (PDF)

S4 Table *cis* and *trans* eQTLs associated with Chromosome 4 SNP rs29210579, p = 4.1x10^-6^. (PDF)

S5 Table *cis* and *trans* eQTLs associated with Chromosome 4 SNP rs29212236, p = 4.1x10^-6^. (PDF)

S6 Table LD blocks surrounding the indicated SNPs using Plink2’s implementation of the Haploview algorithm. (PDF)

## References

[1] Raal FJ, Santos RD, Blom DJ, Marais AD, Charng MJ, Cromwell WC, et al Mipomersen, an apolipoprotein B synthesis inhibitor, for lowering of LDL cholesterol concentrations in patients with homozygous familial hypercholesterolaemia: a randomised, double-blind, placebo-controlled trial. Lancet. 2010;375(9719):998–1006. Epub 2010/03/17. 10.1016/S0140-6736(10)60284-X . 20227758

[2] Jaschinski F, Rothhammer T, Jachimczak P, Seitz C, Schneider A, Schlingensiepen KH. The antisense oligonucleotide trabedersen (AP 12009) for the targeted inhibition of TGF-beta2. Curr Pharm Biotechnol. 2011;12(12):2203–13. Epub 2011/05/31. . 21619536 10.2174/138920111798808266

[3] Al-Asaaed S, Winquist E. Custirsen (OGX-011): clusterin inhibitor in metastatic prostate cancer. Curr Oncol Rep. 2013;15(2):113–8. Epub 2012/12/26. 10.1007/s11912-012-0285-1 . 23266703

[4] Janssen HL, Reesink HW, Lawitz EJ, Zeuzem S, Rodriguez-Torres M, Patel K, et al Treatment of HCV infection by targeting microRNA. N Engl J Med. 2013;368(18):1685–94. Epub 2013/03/29. 10.1056/NEJMoa1209026 . 23534542

[5] Jones NR, Pegues MA, McCrory MA, Singleton W, Bethune C, Baker BF, et al A Selective Inhibitor of Human C-reactive Protein Translation Is Efficacious In Vitro and in C-reactive Protein Transgenic Mice and Humans. Mol Ther Nucleic Acids. 2012;1:e52 Epub 2012/01/01. 10.1038/mtna.2012.44 ; PubMed Central PMCID: PMCPMC3511672. 23629027 PMC3511672

[6] Hua Y, Sahashi K, Rigo F, Hung G, Horev G, Bennett CF, et al Peripheral SMN restoration is essential for long-term rescue of a severe spinal muscular atrophy mouse model. Nature. 2011;478(7367):123–6. Epub 2011/10/08. 10.1038/nature10485 ; PubMed Central PMCID: PMCPMC3191865. 21979052 PMC3191865

[7] Kordasiewicz HB, Stanek LM, Wancewicz EV, Mazur C, McAlonis MM, Pytel KA, et al Sustained therapeutic reversal of Huntington's disease by transient repression of huntingtin synthesis. Neuron. 2012;74(6):1031–44. Epub 2012/06/26. 10.1016/j.neuron.2012.05.009 ; PubMed Central PMCID: PMCPMC3383626. 22726834 PMC3383626

[8] Lee RG, Crosby J, Baker BF, Graham MJ, Crooke RM. Antisense technology: an emerging platform for cardiovascular disease therapeutics. J Cardiovasc Transl Res. 2013;6(6):969–80. Epub 2013/07/17. 10.1007/s12265-013-9495-7 ; PubMed Central PMCID: PMCPMC3838598. 23856914 PMC3838598

[9] Wan WB, Seth PP. The Medicinal Chemistry of Therapeutic Oligonucleotides. J Med Chem. 2016;59(21):9645–67. Epub 2016/07/20. 10.1021/acs.jmedchem.6b00551 . 27434100

[10] Murray S, Ittig D, Koller E, Berdeja A, Chappell A, Prakash TP, et al TricycloDNA-modified oligo-2'-deoxyribonucleotides reduce scavenger receptor B1 mRNA in hepatic and extra-hepatic tissues—a comparative study of oligonucleotide length, design and chemistry. Nucleic Acids Res. 2012;40(13):6135–43. Epub 2012/04/03. 10.1093/nar/gks273 ; PubMed Central PMCID: PMCPMC3401458. 22467214 PMC3401458

[11] Graham MJ, Lee RG, Bell TA 3rd, Fu W, Mullick AE, Alexander VJ, et al Antisense oligonucleotide inhibition of apolipoprotein C-III reduces plasma triglycerides in rodents, nonhuman primates, and humans. Circ Res. 2013;112(11):1479–90. Epub 2013/04/02. 10.1161/CIRCRESAHA.111.300367 . 23542898

[12] van Deventer SJ, Wedel MK, Baker BF, Xia S, Chuang E, Miner PB Jr. A phase II dose ranging, double-blind, placebo-controlled study of alicaforsen enema in subjects with acute exacerbation of mild to moderate left-sided ulcerative colitis. Aliment Pharmacol Ther. 2006;23(10):1415–25. Epub 2006/05/04. 10.1111/j.1365-2036.2006.02910.x . 16669956

[13] Crews KR, Hicks JK, Pui CH, Relling MV, Evans WE. Pharmacogenomics and individualized medicine: translating science into practice. Clin Pharmacol Ther. 2012;92(4):467–75. Epub 2012/09/06. 10.1038/clpt.2012.120 ; PubMed Central PMCID: PMCPMC3589526. 22948889 PMC3589526

[14] Crooke ST. Molecular Mechanisms of Antisense Oligonucleotides. Nucleic acid therapeutics. 2017;27(2):70–7. Epub 2017/01/13. 10.1089/nat.2016.0656 ; PubMed Central PMCID: PMCPMC5372764. 28080221 PMC5372764

[15] White PJ, Anastasopoulos F, Pouton CW, Boyd BJ. Overcoming biological barriers to in vivo efficacy of antisense oligonucleotides. Expert Rev Mol Med. 2009;11:e10 Epub 2009/03/24. 10.1017/S1462399409001021 . 19302730

[16] Altshuler D, Daly MJ, Lander ES. Genetic mapping in human disease. Science. 2008;322(5903):881–8. Epub 2008/11/08. 10.1126/science.1156409 ; PubMed Central PMCID: PMCPMC2694957. 18988837 PMC2694957

[17] Manolio TA. Cohort studies and the genetics of complex disease. Nat Genet. 2009;41(1):5–6. Epub 2008/12/30. 10.1038/ng0109-5 . 19112455

[18] Ghazalpour A, Rau CD, Farber CR, Bennett BJ, Orozco LD, van Nas A, et al Hybrid mouse diversity panel: a panel of inbred mouse strains suitable for analysis of complex genetic traits. Mamm Genome. 2012;23(9–10):680–92. Epub 2012/08/16. 10.1007/s00335-012-9411-5 ; PubMed Central PMCID: PMCPMC3586763. 22892838 PMC3586763

[19] Bennett BJ, Farber CR, Orozco L, Kang HM, Ghazalpour A, Siemers N, et al A high-resolution association mapping panel for the dissection of complex traits in mice. Genome Res. 2010;20(2):281–90. Epub 2010/01/08. 10.1101/gr.099234.109 ; PubMed Central PMCID: PMCPMC2813484. 20054062 PMC2813484

[20] Rau CD, Parks B, Wang Y, Eskin E, Simecek P, Churchill GA, et al High-Density Genotypes of Inbred Mouse Strains: Improved Power and Precision of Association Mapping. G3 (Bethesda). 2015;5(10):2021–6. Epub 2015/08/01. 10.1534/g3.115.020784 ; PubMed Central PMCID: PMCPMC4592984. 26224782 PMC4592984

[21] Hui ST, Parks BW, Org E, Norheim F, Che N, Pan C, et al The genetic architecture of NAFLD among inbred strains of mice. Elife. 2015;4:e05607 Epub 2015/06/13. 10.7554/eLife.05607 ; PubMed Central PMCID: PMCPMC4493743. 26067236 PMC4493743

[22] Farber CR, Bennett BJ, Orozco L, Zou W, Lira A, Kostem E, et al Mouse genome-wide association and systems genetics identify Asxl2 as a regulator of bone mineral density and osteoclastogenesis. PLoS Genet. 2011;7(4):e1002038 Epub 2011/04/15. 10.1371/journal.pgen.1002038 ; PubMed Central PMCID: PMCPMC3072371. 21490954 PMC3072371

[23] Parks BW, Sallam T, Mehrabian M, Psychogios N, Hui ST, Norheim F, et al Genetic architecture of insulin resistance in the mouse. Cell Metab. 2015;21(2):334–46. Epub 2015/02/05. 10.1016/j.cmet.2015.01.002 ; PubMed Central PMCID: PMCPMC4349439. 25651185 PMC4349439

[24] Parks BW, Nam E, Org E, Kostem E, Norheim F, Hui ST, et al Genetic control of obesity and gut microbiota composition in response to high-fat, high-sucrose diet in mice. Cell Metab. 2013;17(1):141–52. Epub 2013/01/15. 10.1016/j.cmet.2012.12.007 ; PubMed Central PMCID: PMCPMC3545283. 23312289 PMC3545283

[25] Rau CD, Wang J, Avetisyan R, Romay MC, Martin L, Ren S, et al Mapping genetic contributions to cardiac pathology induced by Beta-adrenergic stimulation in mice. Circ Cardiovasc Genet. 2015;8(1):40–9. Epub 2014/12/07. 10.1161/CIRCGENETICS.113.000732 ; PubMed Central PMCID: PMCPMC4334708. 25480693 PMC4334708

[26] Kang HM, Zaitlen NA, Wade CM, Kirby A, Heckerman D, Daly MJ, et al Efficient control of population structure in model organism association mapping. Genetics. 2008;178(3):1709–23. Epub 2008/04/04. 10.1534/genetics.107.080101 ; PubMed Central PMCID: PMCPMC2278096. 18385116 PMC2278096

[27] Lippert C, Listgarten J, Liu Y, Kadie CM, Davidson RI, Heckerman D. FaST linear mixed models for genome-wide association studies. Nat Methods. 2011;8(10):833–5. Epub 2011/09/06. 10.1038/nmeth.1681 . 21892150

[28] Miller CM, Donner AJ, Blank EE, Egger AW, Kellar BM, Ostergaard ME, et al Stabilin-1 and Stabilin-2 are specific receptors for the cellular internalization of phosphorothioate-modified antisense oligonucleotides (ASOs) in the liver. Nucleic Acids Res. 2016;44(6):2782–94. Epub 2016/02/26. 10.1093/nar/gkw112 ; PubMed Central PMCID: PMCPMC4824115. 26908652 PMC4824115

[29] Park SY, Yun Y, Lim JS, Kim MJ, Kim SY, Kim JE, et al Stabilin-2 modulates the efficiency of myoblast fusion during myogenic differentiation and muscle regeneration. Nat Commun. 2016;7:10871 Epub 2016/03/15. 10.1038/ncomms10871 ; PubMed Central PMCID: PMCPMC4793076. 26972991 PMC4793076

[30] Lusis AJ, Seldin MM, Allayee H, Bennett BJ, Civelek M, Davis RC, et al The Hybrid Mouse Diversity Panel: a resource for systems genetics analyses of metabolic and cardiovascular traits. J Lipid Res. 2016;57(6):925–42. Epub 2016/04/22. 10.1194/jlr.R066944 ; PubMed Central PMCID: PMCPMC4878195. 27099397 PMC4878195

[31] Jovic M, Kean MJ, Dubankova A, Boura E, Gingras AC, Brill JA, et al Endosomal sorting of VAMP3 is regulated by PI4K2A. J Cell Sci. 2014;127(Pt 17):3745–56. Epub 2014/07/09. 10.1242/jcs.148809 ; PubMed Central PMCID: PMCPMC4150061. 25002402 PMC4150061

[32] Gaus H, Miller CM, Seth PP, Harris EN. Structural Determinants for the Interactions of Chemically Modified Nucleic Acids with the Stabilin-2 Clearance Receptor. Biochemistry. 2018 Epub 2018/03/29. 10.1021/acs.biochem.8b00126 . 29589907 PMC5905987

[33] Heese K, Nakayama T, Hata R, Masumura M, Akatsu H, Li F, et al Characterizing CGI-94 (comparative gene identification-94) which is down-regulated in the hippocampus of early stage Alzheimer's disease brain. Eur J Neurosci. 2002;15(1):79–86. Epub 2002/02/28. . 11860508 10.1046/j.0953-816x.2001.01836.x

[34] Heese K, Nagai Y, Sawada T. Comparative gene identification-94—a pivotal regulator of apoptosis. Neuroscience. 2003;116(2):321–4. Epub 2003/02/01. . 12559088 10.1016/s0306-4522(02)00653-x

[35] Koller E, Vincent TM, Chappell A, De S, Manoharan M, Bennett CF. Mechanisms of single-stranded phosphorothioate modified antisense oligonucleotide accumulation in hepatocytes. Nucleic Acids Res. 2011;39(11):4795–807. Epub 2011/02/25. 10.1093/nar/gkr089 ; PubMed Central PMCID: PMCPMC3113586. 21345934 PMC3113586

[36] Gotoh I, Uekita T, Seiki M. Regulated nucleo-cytoplasmic shuttling of human aci-reductone dioxygenase (hADI1) and its potential role in mRNA processing. Genes Cells. 2007;12(1):105–17. Epub 2007/01/11. 10.1111/j.1365-2443.2006.01035.x . 17212658

[37] Geary RS, Norris D, Yu R, Bennett CF. Pharmacokinetics, biodistribution and cell uptake of antisense oligonucleotides. Adv Drug Deliv Rev. 2015;87:46–51. Epub 2015/02/11. 10.1016/j.addr.2015.01.008 . 25666165

[38] Bennett CF, Swayze EE. RNA targeting therapeutics: molecular mechanisms of antisense oligonucleotides as a therapeutic platform. Annu Rev Pharmacol Toxicol. 2010;50:259–93. Epub 2010/01/09. 10.1146/annurev.pharmtox.010909.105654 . 20055705

[39] Crooke ST, Wang S, Vickers TA, Shen W, Liang XH. Cellular uptake and trafficking of antisense oligonucleotides. Nat Biotechnol. 2017;35(3):230–7. Epub 2017/03/01. 10.1038/nbt.3779 . 28244996

[40] Liang XH, Sun H, Nichols JG, Crooke ST. RNase H1-Dependent Antisense Oligonucleotides Are Robustly Active in Directing RNA Cleavage in Both the Cytoplasm and the Nucleus. Mol Ther. 2017;25(9):2075–92. Epub 2017/07/01. 10.1016/j.ymthe.2017.06.002 ; PubMed Central PMCID: PMCPMC5589097. 28663102 PMC5589097

[41] Donner AJ, Wancewicz EV, Murray HM, Greenlee S, Post N, Bell M, et al Co-Administration of an Excipient Oligonucleotide Helps Delineate Pathways of Productive and Nonproductive Uptake of Phosphorothioate Antisense Oligonucleotides in the Liver. Nucleic acid therapeutics. 2017;27(4):209–20. Epub 2017/04/28. 10.1089/nat.2017.0662 . 28448194

[42] Liang XH, Sun H, Shen W, Crooke ST. Identification and characterization of intracellular proteins that bind oligonucleotides with phosphorothioate linkages. Nucleic Acids Res. 2015;43(5):2927–45. Epub 2015/02/26. 10.1093/nar/gkv143 ; PubMed Central PMCID: PMCPMC4357732. 25712094 PMC4357732

[43] Bailey JK, Shen W, Liang XH, Crooke ST. Nucleic acid binding proteins affect the subcellular distribution of phosphorothioate antisense oligonucleotides. Nucleic Acids Res. 2017;45(18):10649–71. Epub 2017/10/05. 10.1093/nar/gkx709 ; PubMed Central PMCID: PMCPMC5737868. 28977508 PMC5737868

[44] Liang XH, Nichols JG, Sun H, Crooke ST. Translation can affect the antisense activity of RNase H1-dependent oligonucleotides targeting mRNAs. Nucleic Acids Res. 2017 Epub 2017/11/23. 10.1093/nar/gkx1174 . 29165591 PMC5758896

[45] Wang S, Sun H, Tanowitz M, Liang XH, Crooke ST. Intra-endosomal trafficking mediated by lysobisphosphatidic acid contributes to intracellular release of phosphorothioate-modified antisense oligonucleotides. Nucleic Acids Res. 2017;45(9):5309–22. Epub 2017/04/06. 10.1093/nar/gkx231 ; PubMed Central PMCID: PMCPMC5605259. 28379543 PMC5605259

[46] Hung G, Xiao X, Peralta R, Bhattacharjee G, Murray S, Norris D, et al Characterization of target mRNA reduction through in situ RNA hybridization in multiple organ systems following systemic antisense treatment in animals. Nucleic acid therapeutics. 2013;23(6):369–78. Epub 2013/10/29. 10.1089/nat.2013.0443 . 24161045

[47] Nonaka H, Sugano S, Miyajima A. Serial analysis of gene expression in sinusoidal endothelial cells from normal and injured mouse liver. Biochem Biophys Res Commun. 2004;324(1):15–24. Epub 2004/10/07. 10.1016/j.bbrc.2004.09.014 . 15464976

[48] Schmidt K, Prakash TP, Donner AJ, Kinberger GA, Gaus HJ, Low A, et al Characterizing the effect of GalNAc and phosphorothioate backbone on binding of antisense oligonucleotides to the asialoglycoprotein receptor. Nucleic Acids Res. 2017;45(5):2294–306. Epub 2017/02/06. 10.1093/nar/gkx060 ; PubMed Central PMCID: PMCPMC5389643. 28158620 PMC5389643

[49] Ganesan LP, Mates JM, Cheplowitz AM, Avila CL, Zimmerer JM, Yao Z, et al Scavenger receptor B1, the HDL receptor, is expressed abundantly in liver sinusoidal endothelial cells. Sci Rep. 2016;6:20646 Epub 2016/02/13. 10.1038/srep20646 ; PubMed Central PMCID: PMCPMC4749959. 26865459 PMC4749959

[50] Olson MF. Applications for ROCK kinase inhibition. Curr Opin Cell Biol. 2008;20(2):242–8. Epub 2008/02/20. 10.1016/j.ceb.2008.01.002 ; PubMed Central PMCID: PMCPMC2377343. 18282695 PMC2377343

[51] Shi J, Wu X, Surma M, Vemula S, Zhang L, Yang Y, et al Distinct roles for ROCK1 and ROCK2 in the regulation of cell detachment. Cell Death Dis. 2013;4:e483 Epub 2013/02/09. 10.1038/cddis.2013.10 ; PubMed Central PMCID: PMCPMC3734810. 23392171 PMC3734810

[52] Daley WP, Kohn JM, Larsen M. A focal adhesion protein-based mechanochemical checkpoint regulates cleft progression during branching morphogenesis. Dev Dyn. 2011;240(9):2069–83. Epub 2011/10/22. 10.1002/dvdy.22714 ; PubMed Central PMCID: PMCPMC3647453. 22016182 PMC3647453

[53] Ray S, Fanti JA, Macedo DP, Larsen M. LIM kinase regulation of cytoskeletal dynamics is required for salivary gland branching morphogenesis. Mol Biol Cell. 2014;25(16):2393–407. Epub 2014/06/27. 10.1091/mbc.E14-02-0705 ; PubMed Central PMCID: PMCPMC4142612. 24966172 PMC4142612

[54] Wickstrom SA, Fassler R. Regulation of membrane traffic by integrin signaling. Trends Cell Biol. 2011;21(5):266–73. Epub 2011/03/29. 10.1016/j.tcb.2011.02.003 . 21440440

[55] Bridgewater RE, Norman JC, Caswell PT. Integrin trafficking at a glance. J Cell Sci. 2012;125(Pt 16):3695–701. Epub 2012/10/03. 10.1242/jcs.095810 ; PubMed Central PMCID: PMCPMC3462077. 23027580 PMC3462077

[56] Bharadwaj A, Bydoun M, Holloway R, Waisman D. Annexin A2 heterotetramer: structure and function. Int J Mol Sci. 2013;14(3):6259–305. Epub 2013/03/23. 10.3390/ijms14036259 ; PubMed Central PMCID: PMCPMC3634455. 23519104 PMC3634455

[57] Garrido-Gomez T, Dominguez F, Quinonero A, Estella C, Vilella F, Pellicer A, et al Annexin A2 is critical for embryo adhesiveness to the human endometrium by RhoA activation through F-actin regulation. Faseb j. 2012;26(9):3715–27. Epub 2012/05/31. 10.1096/fj.12-204008 . 22645245

[58] Rescher U, Ludwig C, Konietzko V, Kharitonenkov A, Gerke V. Tyrosine phosphorylation of annexin A2 regulates Rho-mediated actin rearrangement and cell adhesion. J Cell Sci. 2008;121(Pt 13):2177–85. Epub 2008/06/21. 10.1242/jcs.028415 . 18565825

[59] Seth PP, Vasquez G, Allerson CA, Berdeja A, Gaus H, Kinberger GA, et al Synthesis and biophysical evaluation of 2',4'-constrained 2'O-methoxyethyl and 2',4'-constrained 2'O-ethyl nucleic acid analogues. J Org Chem. 2010;75(5):1569–81. Epub 2010/02/09. 10.1021/jo902560f . 20136157

[60] Hirose Y, Saijou E, Sugano Y, Takeshita F, Nishimura S, Nonaka H, et al Inhibition of Stabilin-2 elevates circulating hyaluronic acid levels and prevents tumor metastasis. Proc Natl Acad Sci U S A. 2012;109(11):4263–8. Epub 2012/03/01. 10.1073/pnas.1117560109 ; PubMed Central PMCID: PMCPMC3306694. 22371575 PMC3306694

[61] Sehgal A, Vaishnaw A, Fitzgerald K. Liver as a target for oligonucleotide therapeutics. J Hepatol. 2013;59(6):1354–9. Epub 2013/06/19. 10.1016/j.jhep.2013.05.045 . 23770039

[62] Zhang B, Arun G, Mao YS, Lazar Z, Hung G, Bhattacharjee G, et al The lncRNA Malat1 is dispensable for mouse development but its transcription plays a cis-regulatory role in the adult. Cell Rep. 2012;2(1):111–23. Epub 2012/07/31. 10.1016/j.celrep.2012.06.003 ; PubMed Central PMCID: PMCPMC3408587. 22840402 PMC3408587

[63] Yu RZ, Kim TW, Hong A, Watanabe TA, Gaus HJ, Geary RS. Cross-species pharmacokinetic comparison from mouse to man of a second-generation antisense oligonucleotide, ISIS 301012, targeting human apolipoprotein B-100. Drug Metab Dispos. 2007;35(3):460–8. 10.1124/dmd.106.012401 . 17172312

