# Supplementary figures and images for: Mouse genome-wide association studies and systems genetics uncover the genetic architecture associated with hepatic pharmacokinetic and pharmacodynamic properties of a constrained ethyl antisense oligonucleotide targeting Malat1
Source: PLoS Genet. 2018 Oct 29;14(10):e1007732. doi: 10.1371/journal.pgen.1007732 (PMC6224167; doi:10.1371/journal.pgen.1007732)

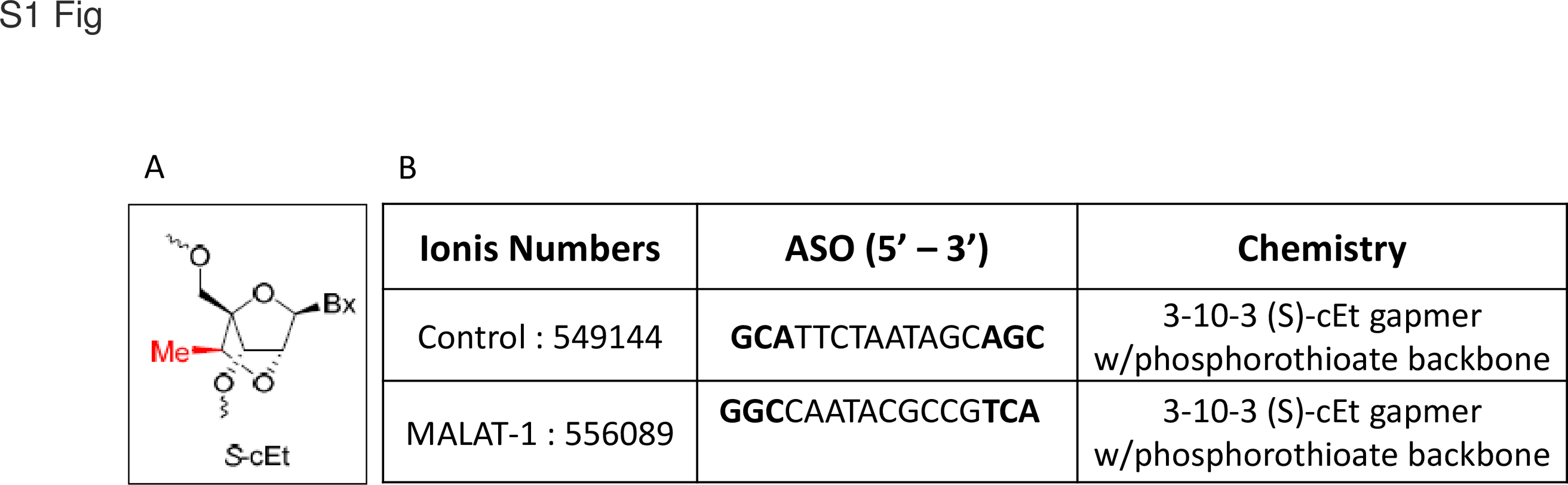

Supplement: S1 Fig — (A) Structural illustration of cET ASOs and PS-MOE ASO showing the organic modifications in the 2’ and 4’ carbon of the ribose moiety and (B) the full length sequence of each specific ASO used in the study. (TIF) [file pgen.1007732.s001.tif]

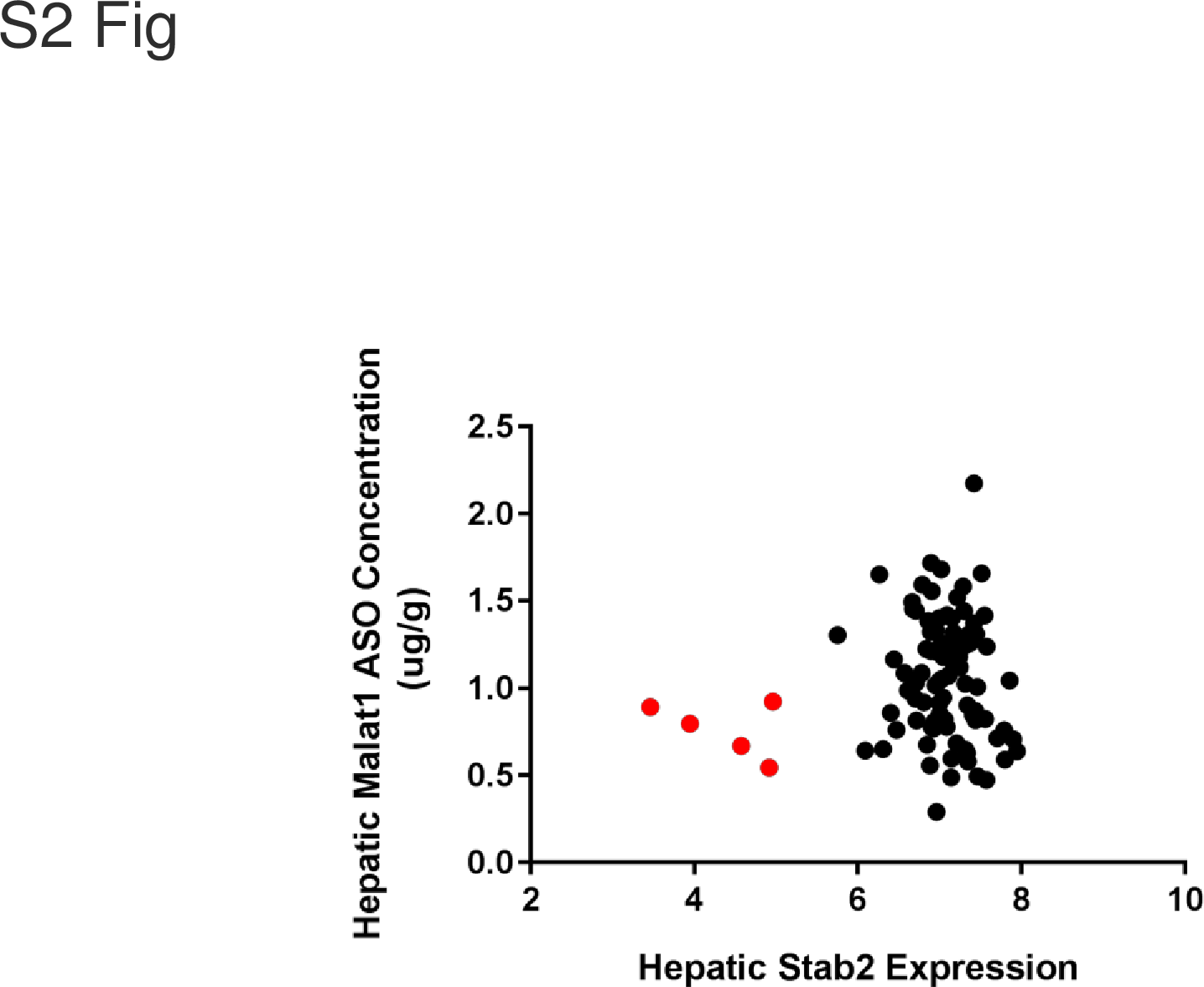

Supplement: S2 Fig — Plot of hepatic Stab2 expression and hepatic accumulation of Malat1 ASO. Red dots indicate the strains with low hepatic Stab2 expression. These outlier strains with low hepatic Stab2 expression were identified. (TIF) [file pgen.1007732.s002.tif]

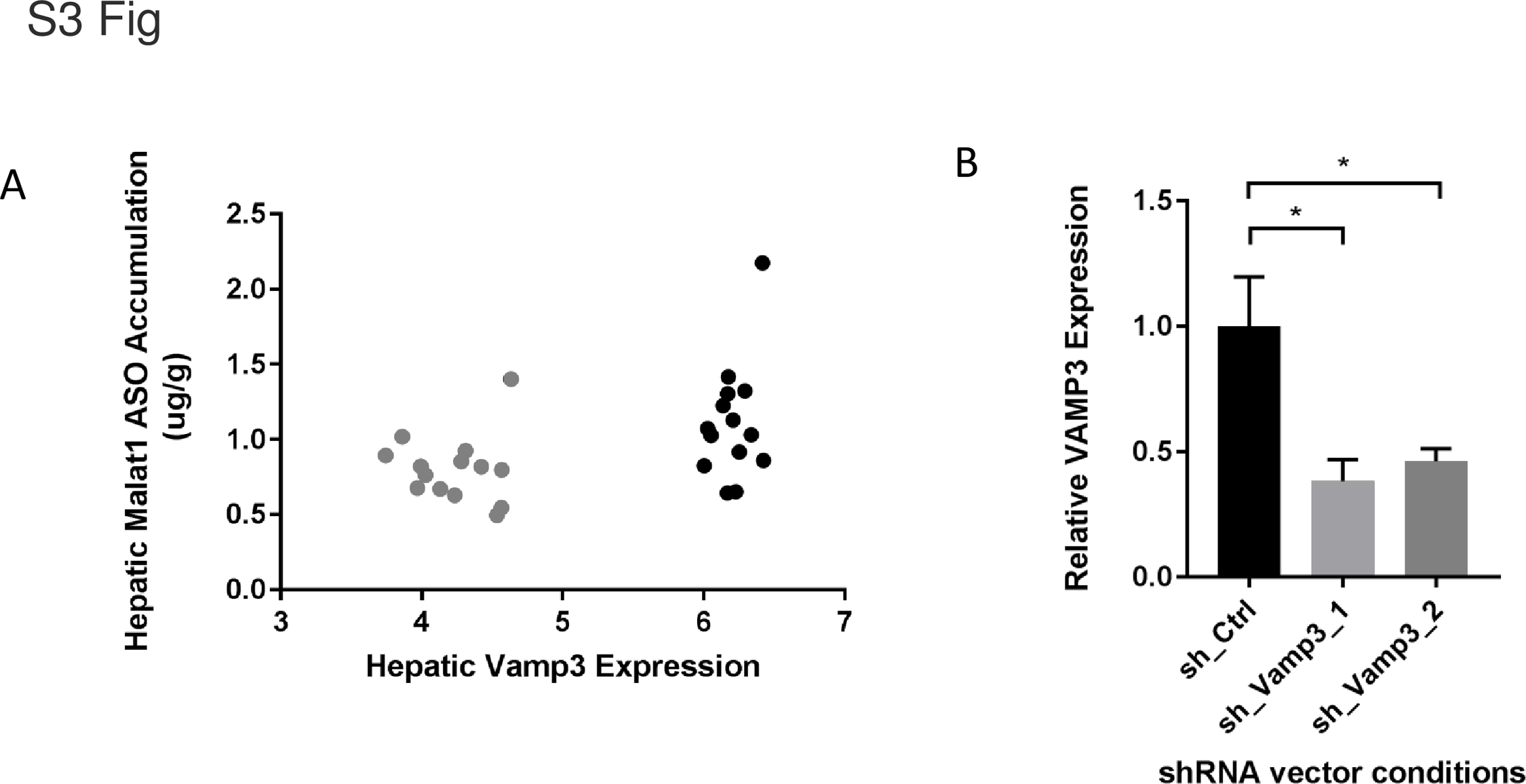

Supplement: S3 Fig — (A) Plot of hepatic Vamp3 expression and hepatic accumulation of Malat1 ASO in BXD strains. Grey dots indicate the strains with low hepatic Vamp3 expression (B) Relative Vamp3 mRNA expression in MHT cells after transduction with either scrambled control or Vamp3 targeting shRNA. Data shows mean ± S.E.M * p ≤ 0.05. (TIF) [file pgen.1007732.s003.tif]

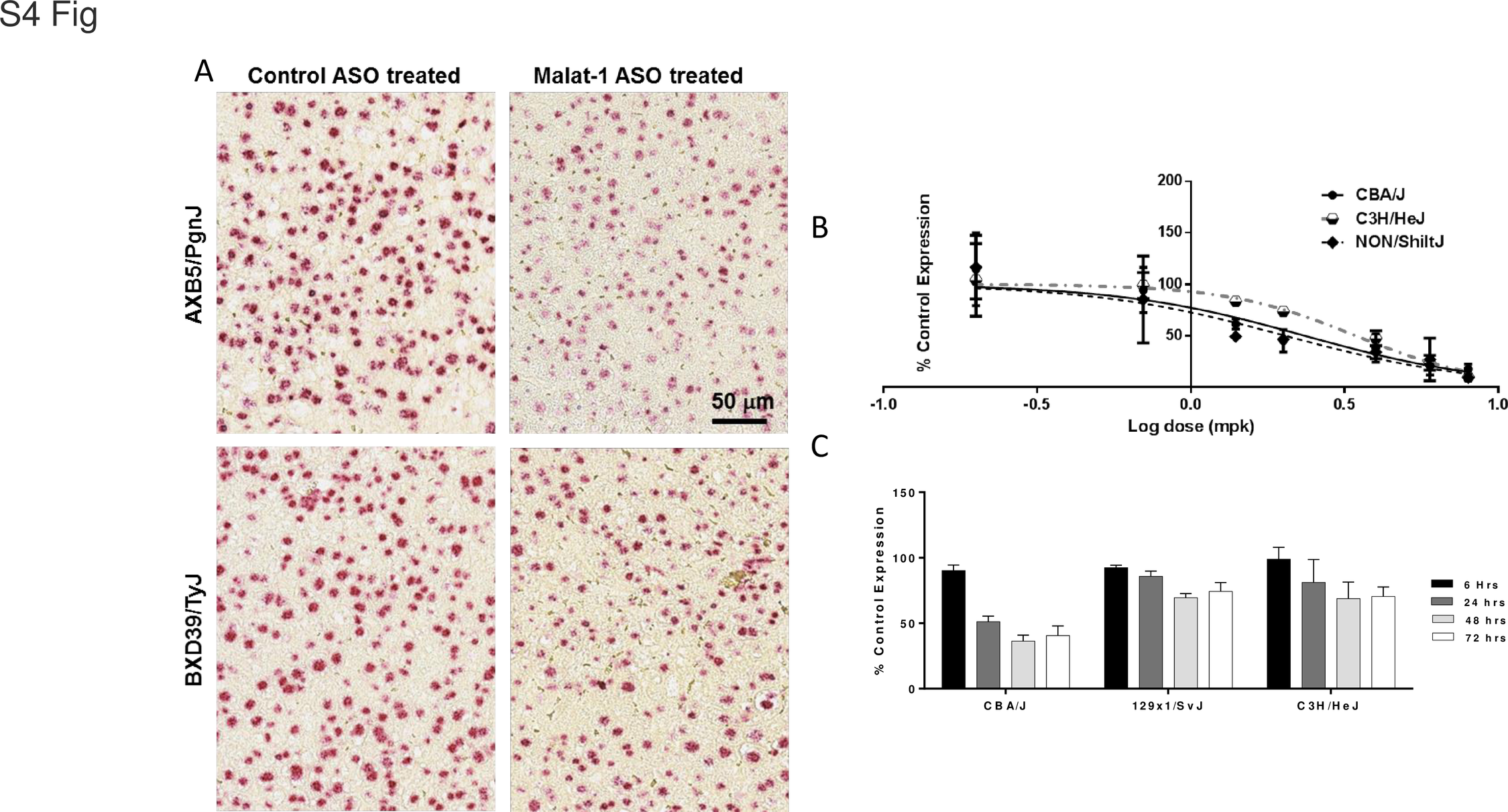

Supplement: S4 Fig — (A) Liver tissue from AXB5/PgnJ and BXD39/TyJ showing Malat1 expression (Malat1 staining is shown in red). AXB5/PgnJ shows a higher reduction Malat1expression than BXD39/ TyJ which is commensurate with hepatic potency of Malat1 ASO in the two strains. Scale = 50 um (B) 72 hour SD dose-response curve in three classic inbred HMDP strains with 0.2, 0.7, 1.4, 2, 4, 6 and 8 mg/kg of Malat1 ASO. (C) Hepatic Malat1 expression in three classic inbred HMDP strains with different time points of incubation (6 hrs, 24 hrs, 48 hrs and 72 hrs) with Malat1 ASO. Data points are mean ± S.E.M. (TIF) [file pgen.1007732.s004.tif]

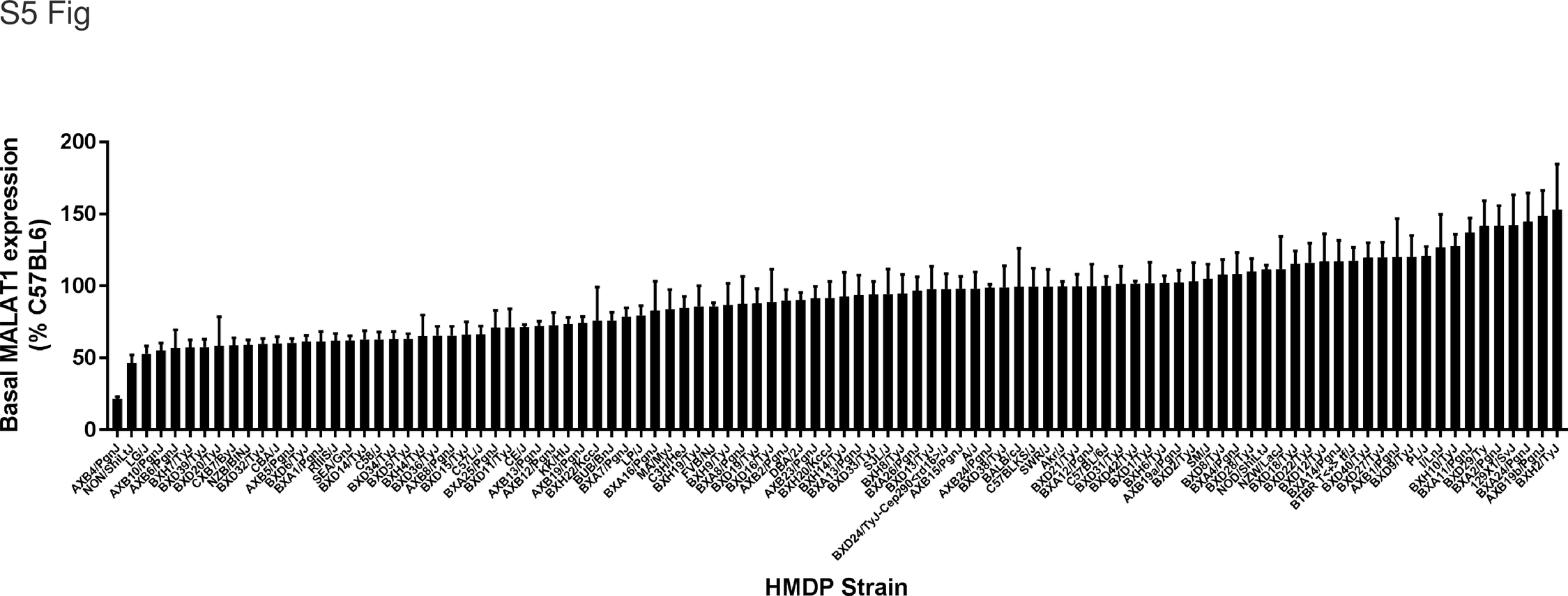

Supplement: S5 Fig — Significant variability was observed in basal hepatic Malat1 expression levels among the 100 strains of HMDP as seen with single 2mg/kg Malat1 ASO dose after 72 hrs. Data points are mean ± S.E.M. (TIF) [file pgen.1007732.s005.tif]

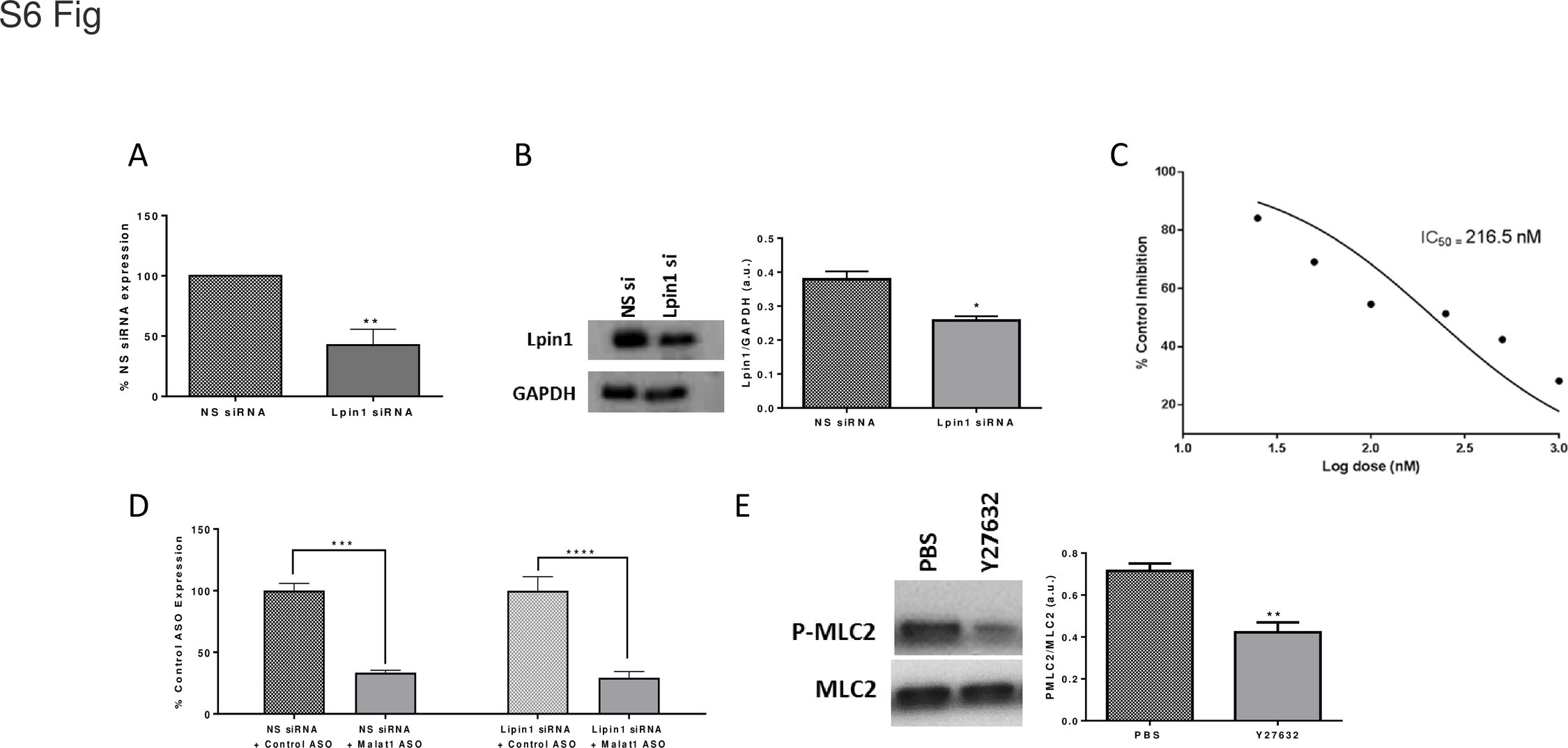

Supplement: S6 Fig — (A) Inhibition of Lpin1 in MHT-1 cells after 48 hours incubation with 32 nM siRNA (B) Western blot analysis to confirm knockdown of Lipin1 (normalized to GAPDH) (C) MHT-1 cells were incubated with 25, 50, 100, 250, 500 and 1000 nM Malat1 ASO for 72 hours. The IC50 was calculated to be 216.5 nM. (D) No significant change in Malat1 ASO potency observed with inhibition of Lpin1 with Lpin1 siRNA as compared to NS siRNA. (E) Western blot analysis confirms downregulation of ROCK activity with significant decrease in pMLC2 levels. Quantification was normalized to MLC2. Data represents mean ± S.E.M. * p ≤ 0.05 ** p ≤ 0.01, *** p ≤ 0.001, **** p ≤ 0.0001, unpaired t-test. (TIF) [file pgen.1007732.s006.tif]

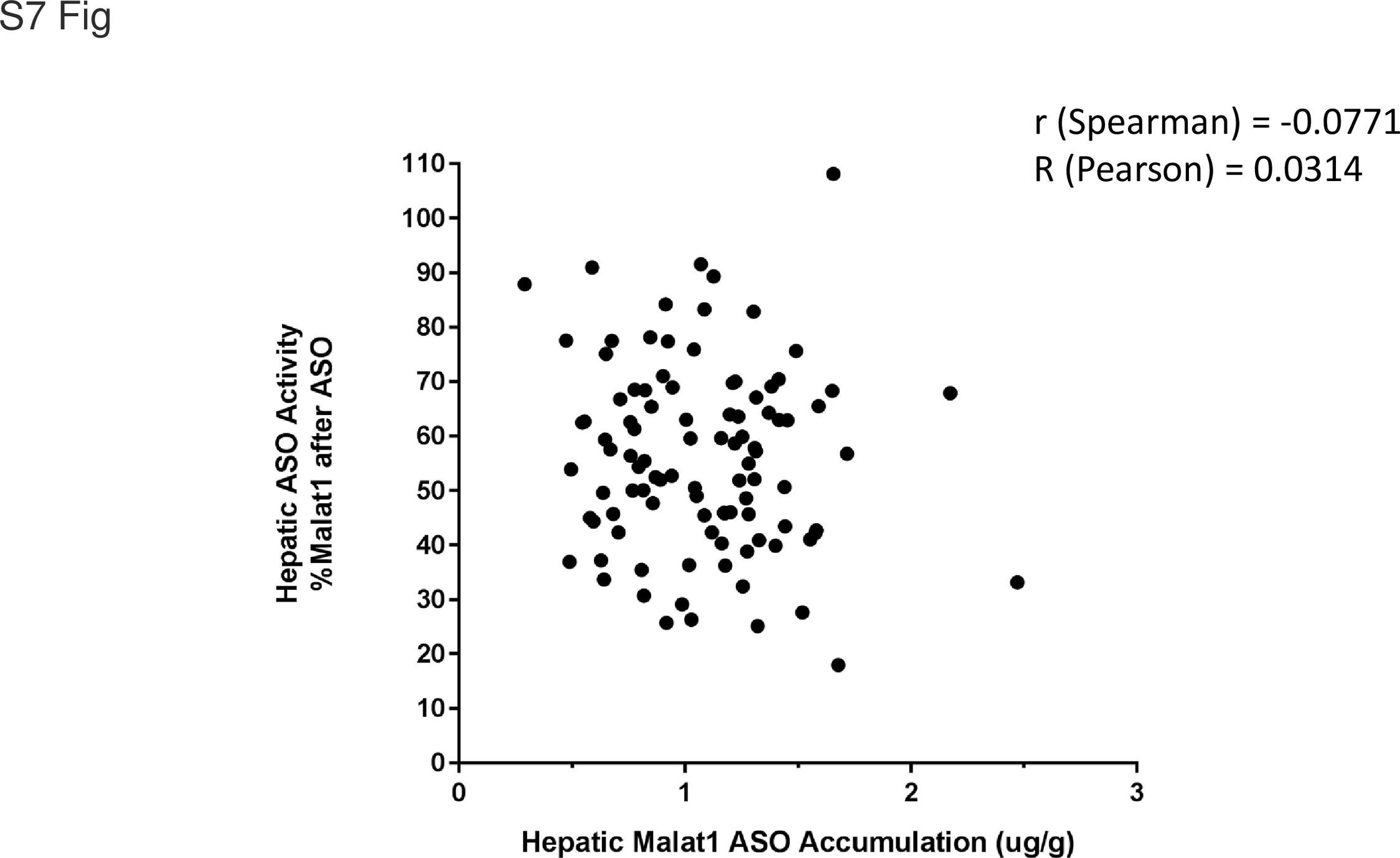

Supplement: S7 Fig — Hepatic potency and accumulation of Malat1 ASO, obtained post single dose of 2mg/kg ASO, in the 100 HMDP strains were correlated. No correlation was observed between hepatic PK and PD of Malat1 ASO for 100 strains. (TIF) [file pgen.1007732.s007.tif]

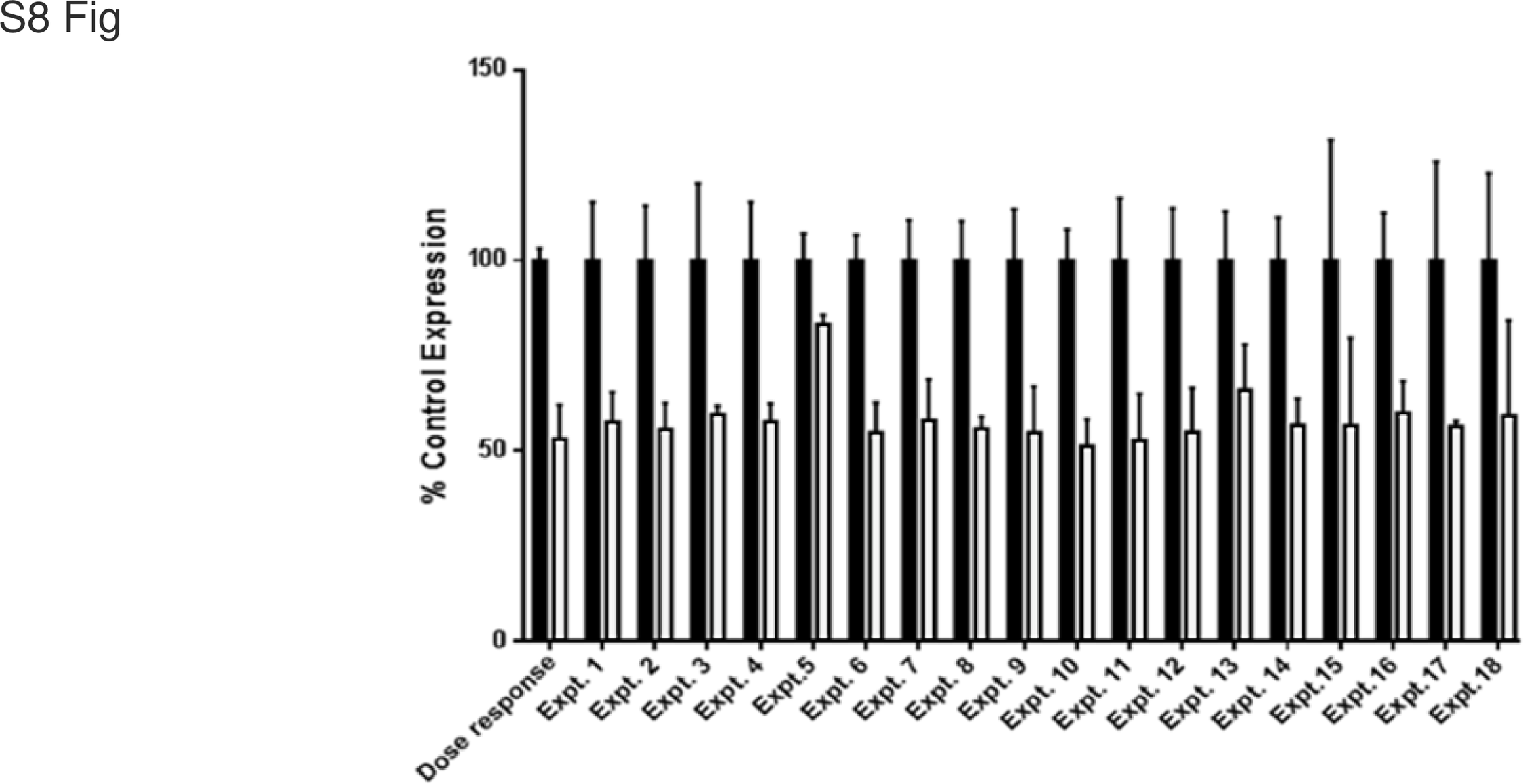

Supplement: S8 Fig — C57BL/6J animals were used in every experiment as a control strain to ensure reproducibility of data. 6 week old male C57/BL/6 animals (n = 4/ strain/treatment) were administered a single dose of 2 mg/kg of ION 556089 (black bar) or ION 549144 (grey bar). After 72 hours, gene expression was assessed in the harvested livers. Similar trends in reduction of hepatic Malat1 expression (normalized to control ASO) were obtained in most of the experiments. Data points are mean ± S.E.M. (TIF) [file pgen.1007732.s008.tif]

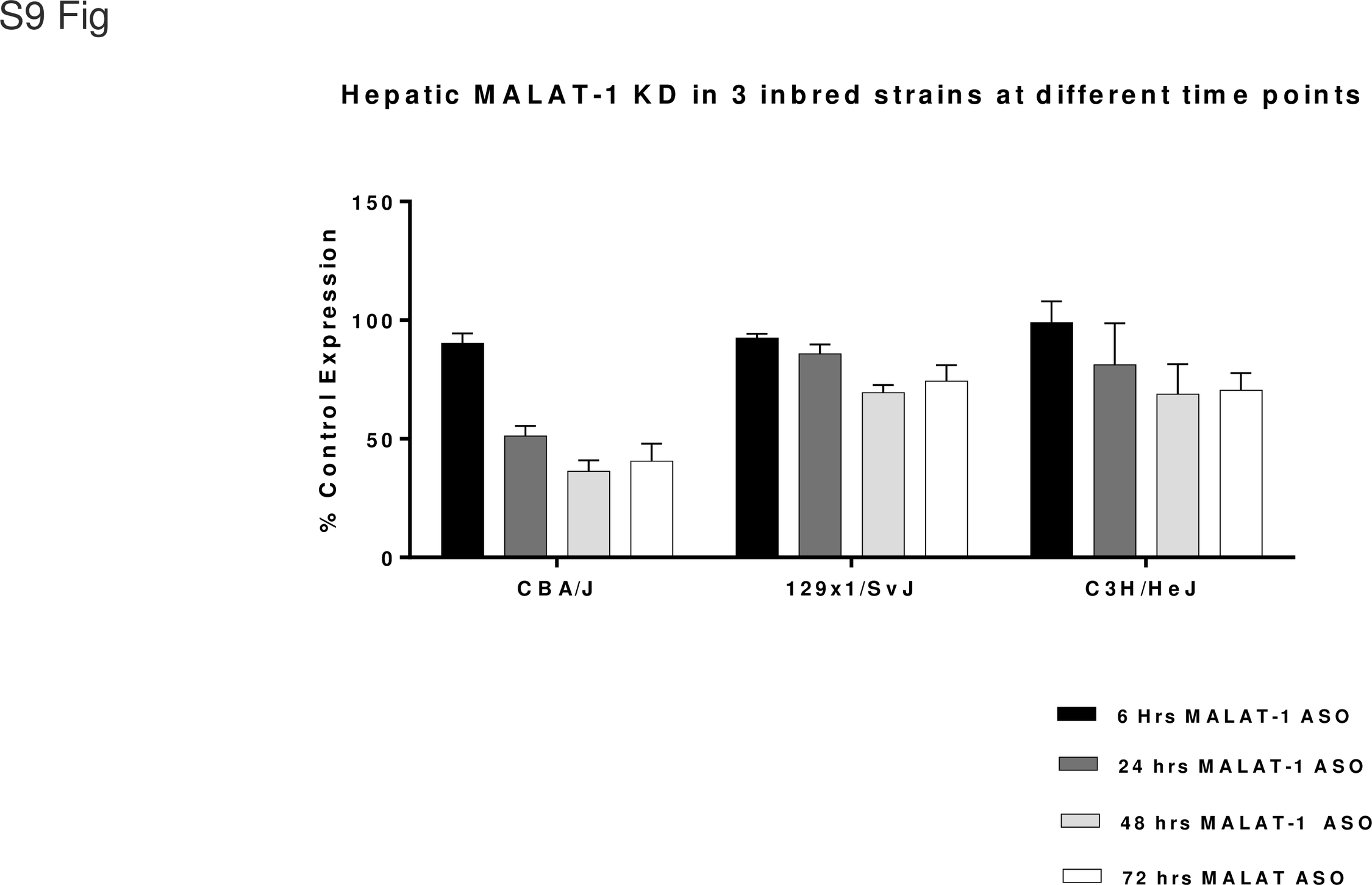

Supplement: S9 Fig — 6 week male mice from Balb/cJ, C57BL6/J and 129x1/SvJ were (n = 5/strain/treatment) were dosed subcutaneously with 1, 3, 5 and 10 mg/kg of either Control cET ASO (ION 549144) or Malat1 cET ASO (ION 556089). Hepatic Malat1 mRNA expression was assessed 72 hours post injection using qRTPCR and results are presented as mean ± S.E.M. (TIF) [file pgen.1007732.s009.tif]

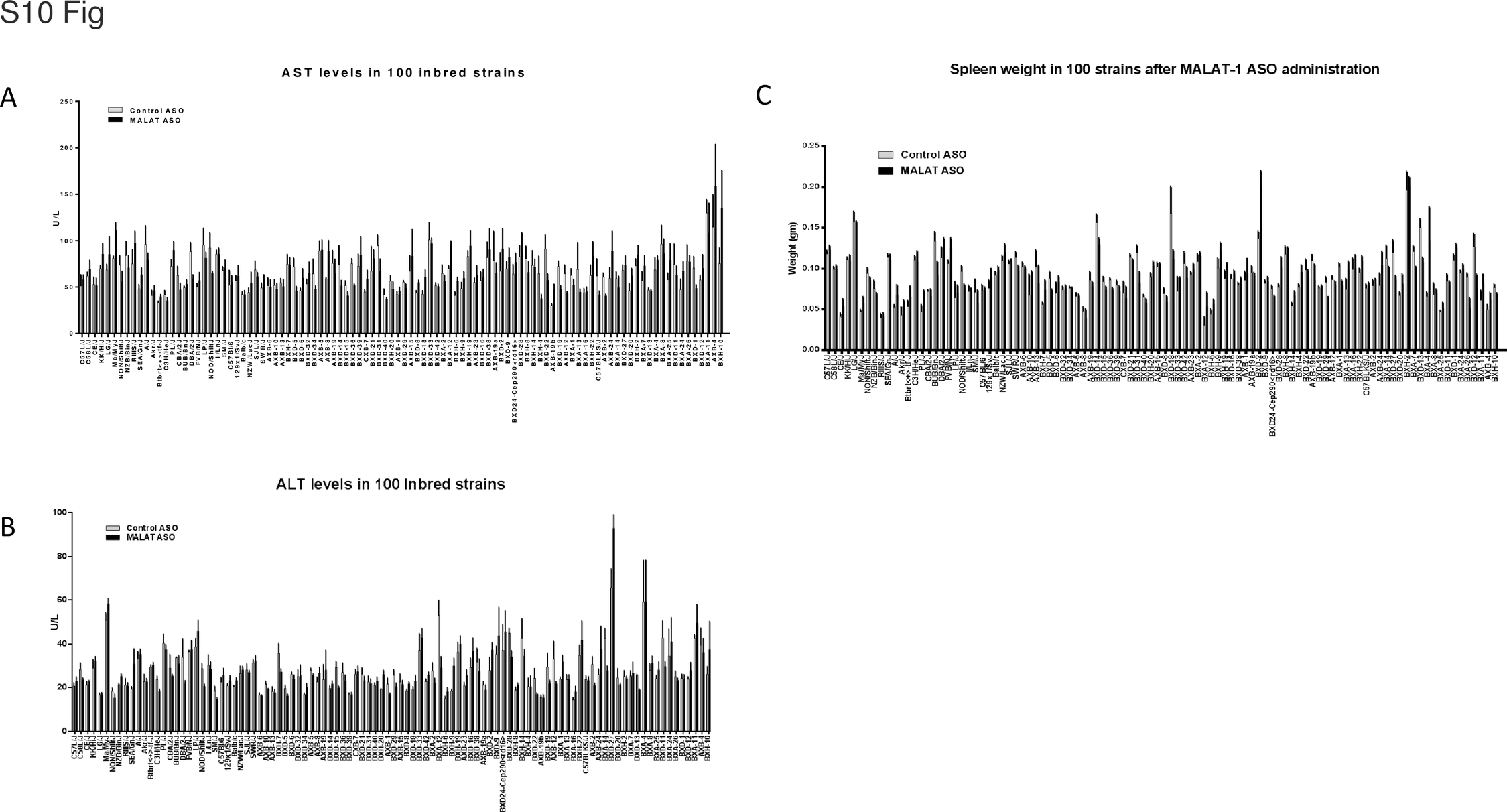

Supplement: S10 Fig — (A) Alanine transaminase (ALT) and Aspartate transaminase (B) levels in serum and (C) spleen weights in the 100 inbred strains after 2mg/kg single dose of Malat1 ASO as compared to Control ASO, 72 hrs post-injection. Data points are mean ± S.E.M. (TIF) [file pgen.1007732.s010.tif]
