# Supplementary material for: Mouse genome-wide association studies and systems genetics uncover the genetic architecture associated with hepatic pharmacokinetic and pharmacodynamic properties of a constrained ethyl antisense oligonucleotide targeting Malat1
Source: PLoS Genet. 2018 Oct 29;14(10):e1007732. doi: 10.1371/journal.pgen.1007732 (PMC6224167; doi:10.1371/journal.pgen.1007732)
Supplement: S1 Table — (PDF) [file pgen.1007732.s011.pdf]

S1 Table

ASO Uptake *cis*-eQTL Chromosome 4 rs32062485

Symbol	Gene Chr.	Gene Name	rsID	p value	Localization
Fam213b	4	family with sequence similarity 213, member B	rs31759536	3.62819E-39	Hepatic
Vamp3	4	vesicle-associated membrane protein 3	rs32925574	2.40505E-28	Hepatic
Ubiad1	4	UbiA prenyltransferase domain containing 1	rs32144125	3.58353E-19	Hepatic
Fv1	4	Friend virus susceptibility 1	rs32925574	3.1979E-16	
Faap20	4	Fanconi anemia core complex associated protein 20	rs32175718	4.7706E-15	Hepatic
Gnb1	4	guanine nucleotide binding protein (G protein), beta 1	rs33102923	9.73864E-14	Hepatic
Mtor	4	mechanistic target of rapamycin (serine/threonine kinase)	rs32925574	3.28275E-12	Hepatic
Tnfrsf14	4	tumor necrosis factor receptor superfamily, member 14	rs33115581	9.83177E-11	Hepatic
Arhgef16	4	Rho guanine nucleotide exchange factor (GEF) 16	rs32671719	2.91791E-09	Hepatic
Tnfrsf1b	4	tumor necrosis factor receptor superfamily, member 1b	rs33111151	4.27209E-09	Hepatic
Exosc10	4	exosome component 10	rs33115456	3.66325E-08	Hepatic
Cep85	4	centrosomal protein 85	rs32773709	2.31819E-07	Hepatic
Spsb1	4	splA/ryanodine receptor domain and SOCS box containing 1	rs32455481	3.2189E-07	Non-Hepatic
Dffa	4	DNA fragmentation factor, alpha subunit	rs32455481	5.49642E-07	Hepatic
Tmem51	4	transmembrane protein 51	rs32925574	1.15705E-06	Hepatic
Pqlc2	4	PQ loop repeat containing 2	rs32925574	1.30411E-06	Hepatic
Foxj3	4	forkhead box J3	rs33081679	1.34699E-06	Hepatic
Hint2	4	histidine triad nucleotide binding protein 2	rs33112359	1.65591E-06	Hepatic

ASO Uptake *trans*-eQTL Chromosome 4 rs32062485

Symbol	Gene Chr.	Gene Name	rsID	p value	Localization
Dvl1	4	dishevelled segment polarity protein 1	rs32185148	2.60159E-34	Hepatic
Gltpd1	4	ceramide-1-phosphate transfer protein	rs32185148	7.05317E-34	Hepatic
Cpsf3l	4	integrator complex subunit 11	rs32185148	4.19846E-21	Hepatic
Akr1e1	13	aldo-keto reductase family 1	rs33109769	1.26642E-15	Hepatic
Ccnl2	4	cyclin L2	rs32185148	1.86876E-15	Hepatic
Mrpl20	4	mitochondrial ribosomal protein L20	rs33102923	1.11055E-14	Hepatic
Agrn	4	agrin	rs32185148	1.37293E-12	Hepatic
Akr1c12	13	aldo-keto reductase family 1, member C12	rs33055189	1.46088E-11	Hepatic
Mxra8	4	matrix-remodelling associated 8	rs31856521	6.29551E-11	Hepatic
Cox6b2	7	cytochrome c oxidase subunit VIb polypeptide 2	rs32987700	2.51768E-10	Non-Hepatic
Aurkaip1	4	aurora kinase A interacting protein 1	rs32867390	4.03152E-10	Hepatic
9430015G10Rik	4	RIKEN cDNA 9430015G10 gene	rs32185148	6.68757E-10	Hepatic
1700020D05Rik	19	RIKEN cDNA 1700020D05	rs33098446	1.05749E-09	
Drap1	19	Dr1 associated protein 1 (negative cofactor 2 alpha)	rs32925574	1.3795E-09	Hepatic
Rap1gap	4	Rap1 GTPase-activating protein	rs33114367	1.80966E-09	Hepatic
Ece1	4	endothelin converting enzyme 1	rs33110013	4.94019E-09	Hepatic
Stk3	15	serine/threonine kinase 3	rs6364274	5.02105E-09	Hepatic
Eif4g3	4	eukaryotic translation initiation factor 4 gamma, 3	rs32747066	1.07142E-08	Hepatic
Gata6	18	GATA binding protein 6	rs33097780	2.72367E-08	Hepatic
Akr1c18	13	aldo-keto reductase family 1, member C18	rs33055189	2.83593E-08	Non-Hepatic
Fam174b	7	family with sequence similarity 174, member B	rs33113373	4.10096E-08	Hepatic
Dnajc24	2	DnaJ heat shock protein family (Hsp40) member C24	rs33064476	6.58198E-08	Hepatic
Mul1	4	mitochondrial ubiquitin ligase activator of NFKB 1	rs33110013	1.37181E-07	Hepatic
Fam63a	3	MINDY lysine 48 deubiquitinase 1	rs33111379	1.45179E-07	Hepatic

Fam114a2	11	family with sequence similarity 114, member A2	rs31759536	1.73688E-07	Hepatic
Stard8	X	START domain containing 8	rs33111151	2.25269E-07	Hepatic
Col5a1	2	collagen, type V, alpha 1	rs33076268	2.62128E-07	Non-Hepatic
C1qtnf4	2	C1q and tumor necrosis factor related protein 4	rs33076170	2.76805E-07	Non-Hepatic
Dnajc28	16	DnaJ heat shock protein family (Hsp40) member C28	rs33060473	4.13988E-07	Hepatic
Rccd1	7	RCC1 domain containing 1	rs33070315	5.48501E-07	Hepatic
Ctns	11	cystinosis, nephropathic	rs33109769	5.89707E-07	Hepatic
Elf3	1	E74-like factor 3	rs33113368	5.97127E-07	Non-Hepatic
Irf7	7	interferon regulatory factor 7	rs32925574	7.36665E-07	Hepatic
1600012H06Rik	17	RIKEN cDNA 1600012H06 gene	rs33111379	7.74073E-07	Hepatic
Fxr1	3	fragile X mental retardation gene 1, autosomal homolog	rs32240565	8.82635E-07	Hepatic
Ptger3	3	prostaglandin E receptor 3 (subtype EP3)	rs33003752	9.87243E-07	Non-Hepatic
Ap3m2	8	adaptor-related protein complex 3, mu 2 subunit	rs33113373	1.36977E-06	Non-Hepatic
Hbegf	18	heparin-binding EGF-like growth factor	rs33109769	1.3746E-06	Non-Hepatic
Atg5	10	autophagy related 5	rs33098446	1.40621E-06	Hepatic
Skp1a	11	S-phase kinase-associated protein 1A	rs32925574	1.4756E-06	Hepatic
Hspa1a	17	heat shock protein 1A	rs33100483	1.81975E-06	
Havcr1	11	hepatitis A virus cellular receptor 1	rs32952372	2.00947E-06	Hepatic
Sh3gl1	17	SH3-domain GRB2-like 1	rs33103096	2.05701E-06	Hepatic
Itpr2	6	inositol 1,4,5-triphosphate receptor 2	rs33111379	2.12799E-06	Hepatic
Slc35a2	X	solute carrier family 35 (UDP-galactose transporter), member A2	rs33103096	2.55659E-06	Hepatic
Snap29	16	inositol 1,4,5-triphosphate receptor 2	rs33077064	2.9029E-06	Hepatic
Cmip	8	c-Maf inducing protein	rs33111379	3.00171E-06	Hepatic
Atad3a	4	ATPase family, AAA domain containing 3A	rs32185148	3.32767E-06	Hepatic
Rpgrip1	14	retinitis pigmentosa GTPase regulator interacting protein 1	rs33077064	3.53459E-06	Hepatic
Slc46a1	11	solute carrier family 46, member 1	rs33106342	3.73866E-06	Hepatic

Pbrm1	14	polybromo 1	rs33070315	3.96851E-06	Hepatic
