# Supplementary material for: Mouse genome-wide association studies and systems genetics uncover the genetic architecture associated with hepatic pharmacokinetic and pharmacodynamic properties of a constrained ethyl antisense oligonucleotide targeting Malat1
Source: PLoS Genet. 2018 Oct 29;14(10):e1007732. doi: 10.1371/journal.pgen.1007732 (PMC6224167; doi:10.1371/journal.pgen.1007732)
Supplement: S2 Table — (PDF) [file pgen.1007732.s012.pdf]

S2 Table

ASO Uptake *cis*-eQTL rs29364476 Chromosome 10

Symbol	Gene Chr.	Gene Name	rsID	p value	Localization
Nedd1	10	neural precursor cell expressed, developmentally down-regulated gene 1	rs6222436	6.87707E-18	Hepatic
Ndufa12	10	NADH dehydrogenase (ubiquinone) 1 alpha subcomplex, 12	rs50617055	3.09593E-15	Hepatic
Nfyb	10	nuclear transcription factor-Y beta	rs29349626	6.43459E-11	Non-Hepatic
Amdhd1	10	amidohydrolase domain containing 1	rs36924269	2.849E-09	Hepatic
Uhrf1bp1l	10	UHRF1 (ICBP90) binding protein 1-like	rs29353578	1.22142E-08	Hepatic
Nt5dc3	10	5'-nucleotidase domain containing 3	rs29320622	1.50441E-08	Hepatic
Fig4	10	FIG4 phosphoinositide 5-phosphatase	rs51355169	3.637E-07	Non-Hepatic
Pah	10	phenylalanine hydroxylase	rs29348009	1.54626E-06	Hepatic
Stab2	10	stabilin 2	rs30243927	3.48204E-06	Hepatic

ASO Uptake *trans*-eQTL rs29364476 Chromosome 10

Symbol	Gene Chr.	Gene Name	rsID	p value	Localization
Hbegf	18	heparin-binding EGF-like growth factor	rs51355169	1.90735E-16	Non-Hepatic
Lta4h	10	leukotriene A4 hydrolase	rs50847752	1.72835E-12	Hepatic
Actr6	10	ARP6 actin-related protein 6	rs29341703	3.16256E-12	Hepatic
Sepw1	7	selenoprotein W	rs30242163	3.38427E-12	Hepatic
Mmp12	9	matrix metallopeptidase 12	rs51355169	8.68859E-10	Non-Hepatic
Casp9	4	caspase 9	rs51355169	4.98257E-09	Hepatic
Fmod	1	fibromodulin	rs51355169	1.23495E-08	Hepatic
Fmn1	2	formin 1	rs51355169	3.41644E-08	Hepatic
Clcn7	17	chloride channel, voltage-sensitive 7	rs51355169	6.16013E-08	Hepatic
Vamp3	4	vesicle-associated membrane protein 3	rs29315879	6.32066E-08	Hepatic
Clcn3	8	chloride channel, voltage-sensitive 3	rs30242163	7.32935E-08	Hepatic
Vps4b	1	vacuolar protein sorting 4B	rs30246486	9.35167E-08	Hepatic
Ackr2	9	atypical chemokine receptor 2	rs51355169	9.63891E-08	Hepatic
Pja1	X	praja ring finger ubiquitin ligase 1	rs30241858	1.14002E-07	Hepatic
Kcne3	7	potassium voltage-gated channel, Isk-related subfamily, gene 3	rs29371234	2.00084E-07	Non-Hepatic
D19Bwg1357e	19	pumilio RNA-binding family member 3	rs50412470	4.59607E-07	Hepatic
Psme4	11	proteasome (prosome, macropain) activator subunit 4	rs51355169	4.67172E-07	Hepatic
Tulp1	17	tubby like protein 1	rs51355169	5.29485E-07	Non-Hepatic
Rgs10	7	regulator of G-protein signalling 10	rs51355169	6.13397E-07	Hepatic
Akr1e1	13	aldo-keto reductase family 1, member E1	rs13480690	7.30781E-07	Hepatic
Dqx1	6	DEAQ RNA-dependent ATPase	rs30241858	8.24512E-07	Hepatic
Fbxw11	11	F-box and WD-40 domain protein 11	rs51355169	8.94646E-07	Hepatic
Uba2	7	ubiquitin-like modifier activating enzyme 2	rs51355169	1.14828E-06	Hepatic
H1fx	6	H1 histone family, member X	rs51355169	1.21804E-06	Hepatic

Nol7	13	nucleolar protein 7	rs51355169	1.2357E-06	Hepatic
Alox12	11	arachidonate 12-lipoxygenase	rs51355169	1.25733E-06	Hepatic
Mrpl35	6	mitochondrial ribosomal protein L35	rs51355169	1.53695E-06	Hepatic
Vps29	5	VPS29 retromer complex component	rs51355169	2.19758E-06	Hepatic
Pign	1	phosphatidylinositol glycan anchor biosynthesis, class N	rs13480690	2.28145E-06	Hepatic
Armt1	10	acidic residue methyltransferase 1	rs29335877	2.61239E-06	Hepatic
Hipk3	2	homeodomain interacting protein kinase 3	rs52052267	2.63859E-06	Hepatic
Pdha1	X	pyruvate dehydrogenase E1 alpha 1	rs45715710	3.30902E-06	Hepatic
Ubr5	15	ubiquitin protein ligase E3 component n-recognin 5	rs13480690	3.32605E-06	Hepatic
Cdca4	12	cell division cycle associated 4	rs29379178	3.42941E-06	Hepatic
Cnn1	9	calponin 1	rs51355169	3.79509E-06	Non-Hepatic
Irak1	X	interleukin-1 receptor-associated kinase 1	rs51355169	3.87193E-06	Hepatic
