# Supplementary material for: Mouse genome-wide association studies and systems genetics uncover the genetic architecture associated with hepatic pharmacokinetic and pharmacodynamic properties of a constrained ethyl antisense oligonucleotide targeting Malat1
Source: PLoS Genet. 2018 Oct 29;14(10):e1007732. doi: 10.1371/journal.pgen.1007732 (PMC6224167; doi:10.1371/journal.pgen.1007732)
Supplement: S3 Table — (PDF) [file pgen.1007732.s013.pdf]

S3 Table

ASO Activity *cis*-eQTL Chromosome 4 rs27549337

Symbol	Gene Chr.	Gene Name	rsID	p value	Localization
Ncdn	4	Neurochondrin	rs27549413	2.03E-08	Non-Hepatic
Tceb3	4	Transcription elongation factor B polypeptide 3	rs27549413	4.23E-08	Hepatic
Utp11l	4	Small Subunit Processome Component	rs32899752	1.02E-07	Hepatic
Hdac1	4	Histone Deacetylase 1	rs27549240	4.39E-07	Hepatic

ASO Activity *trans*-eQTL Chromosome 4 rs27549337

Symbol	Gene Chr.	Gene Name	rsID	p value	Localization
Cap1	4	CAP, adenylate cyclase-associated protein 1	rs27551429	2.39689E-19	Hepatic
Tmem234	4	transmembrane protein 234	rs32857846	6.95986E-18	Hepatic
Hspa1a	17	heat shock protein 1A	rs27547838	1.09381E-13	
Mul1	4	mitochondrial ubiquitin ligase activator of NFKB 1	rs27566735	8.96351E-13	Hepatic
Yae1d1	13	Yae1 domain containing 1	rs27566718	9.08787E-12	Hepatic
Fam167b	4	Fam167	rs3704331	1.3168E-09	Hepatic
Sytl1	4	synaptotagmin-like 1	rs32381686	2.32881E-09	Non-Hepatic
Hp1bp3	4	heterochromatin protein 1, binding protein 3	rs27563778	3.0088E-09	Hepatic
Foxj3	4	forkhead box J3	rs27566735	4.27993E-09	Hepatic
Alpl	4	Alpl	rs27522398	8.36997E-09	Hepatic
Ece1	4	endothelin converting enzyme 1	rs32989901	2.85682E-08	Hepatic
Ptp4a2	4	protein tyrosine phosphatase 4a2	rs27551429	4.76135E-08	Hepatic
Rps6ka1	4	ribosomal protein S6 kinase polypeptide 1	rs27551429	5.17814E-08	Hepatic
Csnk2a1	2	casein kinase 2, alpha 1 polypeptid	rs27566718	5.27057E-08	Hepatic
Ppt1	4	palmitoyl-protein thioesterase 1	rs27565901	7.26142E-08	Hepatic
Dffa	4	DNA fragmentation factor, alpha subunit	rs27566622	7.51459E-08	Hepatic
Gstp3	19	glutathione S-transferase pi 3	rs27550732	9.47912E-08	Hepatic
Pqlc2	4	PQ loop repeat containing 2	rs32979115	9.57617E-08	Hepatic
Tmem82	4	transmembrane protein 82	rs27549240	1.00507E-07	Hepatic

Wdtc1	4	WD and tetratricopeptide repeats 1	rs27566735	1.47796E-07	Hepatic
Rad51b	12	RAD51 paralog B	rs27526591	1.73763E-07	Hepatic
Adprhl2	4	ADP-ribosylhydrolase like 2	rs27547982	1.79333E-07	Hepatic
Igbp1	X	immunoglobulin (CD79A) binding protein 1	rs27549331	1.87519E-07	Hepatic
Cgnl1	9	cingulin-like 1	rs27528887	2.02079E-07	Hepatic
Serpina3a	12	serine (or cysteine) peptidase inhibitor, clade A, member 3A	rs27526591	2.03761E-07	Non-Hepatic
Cap2	13	CAP, adenylate cyclase-associated protein, 2	rs27526591	2.03796E-07	Non-Hepatic
AW209491	13	expressed sequence AW209491	rs27532503	2.57316E-07	Hepatic
Stmn1	4	stathmin 1	rs27528639	2.84614E-07	Non-Hepatic
1700020D05Rik	19	RIKEN cDNA 1700020D05 gene	rs27550732	3.02657E-07	
Alas2	X	aminolevulinic acid synthase 2, erythroid	rs27564633	3.26942E-07	Hepatic
Eif4g3	4	eukaryotic translation initiation factor 4 gamma, 3	rs32979115	3.33081E-07	Hepatic
Cml2	6	N-acetyltransferase 8 (GCN5-related) family member 2	rs27528887	3.4159E-07	Hepatic
Lsm10	4	U7 snRNP-specific Sm-like protein LSM10	rs27550131	3.4853E-07	Hepatic
Slc17a2	13	solute carrier family 17 (sodium phosphate), member 2	rs27526591	6.99514E-07	Hepatic
Pex11a	7	peroxisomal biogenesis factor 11 alpha	rs27566622	7.74313E-07	Hepatic
Stim1	7	stromal interaction molecule 1	rs27528887	7.77024E-07	Hepatic
Natd1	11	N-acetyltransferase domain containing 1	rs27529025	8.53093E-07	Hepatic
Angel2	1	angel homolog 2	rs27529021	8.92921E-07	Hepatic
Extl1	4	exostoses (multiple)-like 1	rs27578338	9.45797E-07	Hepatic
Nnt	13	nicotinamide nucleotide transhydrogenase	rs27581005	1.10297E-06	Hepatic
Tdp2	13	tyrosyl-DNA phosphodiesterase 2	rs27526591	1.18188E-06	Hepatic
P4ha1	10	procollagen-proline, 2-oxoglutarate 4-dioxygenase (proline 4-hydroxylase), alpha 1 polypeptide	rs27526591	1.50741E-06	Hepatic
Smc4	3	structural maintenance of chromosomes 4	rs27532471	1.60664E-06	Non-Hepatic
Gbas	5	nipsnap homolog 2	rs27578608	1.66812E-06	Hepatic

Scnn1g	7	sodium channel, nonvoltage-gated 1 gamma	rs27566215	1.7595E-06	Non-Hepatic
Pvr	7	poliovirus receptor	rs27566622	1.76516E-06	Hepatic
Chka	19	choline kinase alpha	rs27581005	2.40907E-06	Hepatic
Smox	2	spermine oxidase	rs27532388	2.91947E-06	Non-Hepatic
Cenpc1	5	centromere protein C1	rs27528887	3.0287E-06	Non-Hepatic
Mut	17	methylmalonyl-Coenzyme A mutase	rs27532664	3.10966E-06	Hepatic
Mapk7	11	mitogen-activated protein kinase 7	rs27549748	3.27386E-06	Hepatic
Mthfd2	6	methylenetetrahydrofolate dehydrogenase (NAD+ dependent), methenyltetrahydrofolate cyclohydrolase	rs27550793	0.000003329	Non-Hepatic
Ltbp1	17	latent transforming growth factor beta binding protein 1	rs27526429	3.59453E-06	Non-Hepatic
Eif3a	19	eukaryotic translation initiation factor 3, subunit A	rs27551286	3.71956E-06	Hepatic
Gpbp1l1	4	GC-rich promoter binding protein 1-like 1	rs27549413	3.73756E-06	Hepatic
Exoc7	11	exocyst complex component 7	rs27563753	3.87456E-06	Hepatic
Mthfr	4	methylenetetrahydrofolate reductase	rs27581005	3.94153E-06	Hepatic
Tceanc2	4	transcription elongation factor A (SII) N-terminal and central domain containing 2	rs27551429	4.06448E-06	Hepatic
Lenep	3	lens epithelial protein	rs27528933	4.09754E-06	Hepatic
