# Supplementary material for: Mouse genome-wide association studies and systems genetics uncover the genetic architecture associated with hepatic pharmacokinetic and pharmacodynamic properties of a constrained ethyl antisense oligonucleotide targeting Malat1
Source: PLoS Genet. 2018 Oct 29;14(10):e1007732. doi: 10.1371/journal.pgen.1007732 (PMC6224167; doi:10.1371/journal.pgen.1007732)
Supplement: S4 Table — (PDF) [file pgen.1007732.s014.pdf]

S4 Table

ASO Activity *cis*-eQTL Chromosome 10 rs29210579

Gene Symbol	Gene Chr.	Gene Name	rsID	P value	Localization
Iah1	12	isoamyl acetate-hydrolyzing esterase 1 homolog	rs48369508	1.58127E-61	Hepatic
Adi1	12	acireductone dioxygenase 1	rs45838918	7.31225E-27	Hepatic
E2f6	12	E2F transcription factor 6	rs3684777	6.21236E-24	Hepatic
Lpin1	12	lipin 1	rs49484171	9.17205E-22	Hepatic
Trappc12	12	trafficking protein particle complex 12	rs29144264	2.40415E-21	Hepatic
Acp1	12	acid phosphatase 1, soluble	rs49260494	1.18094E-20	Hepatic
Rrm2	12	ribonucleotide reductase M2	rs29155982	4.0135E-17	Hepatic
Smc6	12	structural maintenance of chromosomes 6	rs29490142	2.3226E-11	Hepatic
Itgb1bp1	12	integrin beta 1 binding protein 1	rs47211551	3.20623E-11	Hepatic
Rock2	12	Rho-associated coiled-coil containing protein kinase 2	rs46223186	1.27152E-10	Hepatic
Cpsf3	12	cleavage and polyadenylation specificity factor 3	rs29184145	2.3677E-10	Hepatic
Hpcal1	12	hippocalcin-like 1	rs45935031	1.14569E-09	Hepatic
Cys1	12	cystin 1	rs47354965	1.71308E-09	Non-Hepatic
Zfp277	12	zinc finger protein 277	rs45838918	3.29896E-09	Hepatic
Rdh14	12	retinol dehydrogenase 14	rs48623130	2.37663E-08	Hepatic
Cbll1	12	Casitas B-lineage lymphoma-like 1provided	rs51175945	3.01035E-08	Hepatic
Taf1b	12	TATA-box binding protein associated factor, RNA polymerase I, B	rs47487450	3.46195E-08	Hepatic
Pdia6	12	protein disulfide isomerase associated 6	rs50683287	1.8096E-07	Hepatic
Adam17	12	a disintegrin and metallopeptidase domain 17	rs3684777	5.85721E-07	Hepatic
Sypl	12	synaptophysin-like protein	rs13481321	5.99387E-07	Hepatic
Fos	12	FBJ osteosarcoma oncogene	rs46261446	1.74995E-06	Non-Hepatic
Sntg2	12	syntrophin, gamma 2	rs29169538	1.76514E-06	Hepatic

ASO Activity *trans*-eQTL Chromosome 10 rs29210579

Gene Symbol	Gene Chr.	Gene Name	rsID	P value	Localization
Nat8f4	6	N-acetyltransferase 8 (GCN5-related) family member 4	rs29488295	6.08373E-10	Hepatic
Sdc4	2	syndecan 4	rs47938088	1.8318E-07	Hepatic
Gdpd3	7	glycerophosphodiester phosphodiesterase domain containing 3	rs45935031	2.02673E-07	Hepatic
Isoc2b	7	isochorismatase domain containing 2b	rs49480456	2.28258E-07	Hepatic
Prkag1	15	protein kinase, AMP-activated, gamma 1 non-catalytic subunit	rs29223833	4.71276E-07	Hepatic
Lnx1	5	ligand of numb-protein X 1	rs48339803	5.24089E-07	Hepatic
BC003331	1	cDNA sequence BC003331	rs49366144	5.36808E-07	Hepatic
1700020D05Rik	19	RIKEN cDNA 1700020D05 gene	rs46261446	9.96558E-07	unknown
Akr1c12	13	aldo-keto reductase family 1, member C12	rs46261446	1.40275E-06	Hepatic
Dsg2	18	desmoglein 2	rs33844632	1.51741E-06	Hepatic
Hint2	4	histidine triad nucleotide binding protein 2	rs47938088	1.69072E-06	Hepatic
Rnf34	5	ring finger protein 34	rs47980109	1.76689E-06	Hepatic
Wnt2	6	wingless-type MMTV integration site family, member 2	rs3023949	1.79696E-06	Hepatic
Nnt	13	nicotinamide nucleotide transhydrogenase	rs13481324	1.94054E-06	Hepatic
Mthfd2	6	methylenetetrahydrofolate dehydrogenase (NAD+ dependent), methenyltetrahydrofolate cyclohydrolase	rs47452109	2.02734E-06	Non-Hepatic
Reep3	10	receptor accessory protein 3	rs49744833	3.08674E-06	Hepatic
Aktip	8	thymoma viral proto-oncogene 1 interacting protein	rs29166030	3.24034E-06	Hepatic
Pbld2	10	phenazine biosynthesis-like protein domain containing 2	rs48339803	3.64573E-06	Hepatic
Rpap2	5	RNA polymerase II associated protein 2	rs29124559	3.80246E-06	Hepatic
Myom2	8	myomesin 2	rs46261446	3.84867E-06	Non-Hepatic
Vamp1	6	vesicle-associated membrane protein 1	rs47818728	4.04978E-06	Hepatic
