# Supplementary material for: Mouse genome-wide association studies and systems genetics uncover the genetic architecture associated with hepatic pharmacokinetic and pharmacodynamic properties of a constrained ethyl antisense oligonucleotide targeting Malat1
Source: PLoS Genet. 2018 Oct 29;14(10):e1007732. doi: 10.1371/journal.pgen.1007732 (PMC6224167; doi:10.1371/journal.pgen.1007732)
Supplement: S5 Table — (PDF) [file pgen.1007732.s015.pdf]

S5 Table

ASO Activity *cis*-eQTL Chromosome 10 rs29212236

Gene Symbol	Gene Chr.	Gene Name	rsID	P value	Localization
Adi1	12	acireductone dioxygenase 1	rs47318213	3.40285E-38	Hepatic
Iah1	12	isoamyl acetate-hydrolyzing esterase 1	rs48414422	6.12146E-31	Hepatic
Trappc12	12	trafficking protein particle complex 12	rs29205864	2.32452E-28	Hepatic
Acp1	12	acid phosphatase 1	rs51659732	2.04979E-22	Hepatic
E2f6	12	E2F transcription factor 6	rs48058564	3.35929E-22	Non-Hepatic
Lpin1	12	lipin 1	rs29159678	1.78584E-20	Hepatic
Rrm2	12	ribonucleotide reductase M2	rs30768905	7.15625E-17	Hepatic
Zfp277	12	zinc finger protein 277	rs47318213	1.05931E-12	Hepatic
Smc6	12	structural maintenance of chromosomes 6	rs29485826	2.60259E-12	Hepatic
Cys1	12	cystin 1	rs31635405	3.2693E-12	Non-Hepatic
Cpsf3	12	cleavage and polyadenylation specificity factor 3	rs46095353	2.72473E-11	Hepatic
Rock2	12	Rho-associated coiled-coil containing protein kinase 2	rs31626421	1.41997E-09	Hepatic
Cbll1	12	Casitas B-lineage lymphoma-like 1provided	rs31635405	1.65245E-09	Hepatic
Sntg2	12	syntrophin, gamma 2	rs29179106	1.03864E-08	Non-Hepatic
Taf1b	12	TATA-box binding protein associated factor, RNA polymerase I, B	rs47075250	7.50314E-08	Hepatic
Sypl	12	synaptophysin-like protein	rs31635405	1.30719E-07	Hepatic
Rdh14	12	retinol dehydrogenase 14	rs52225319	3.3898E-07	Hepatic
Adam17	12	a disintegrin and metallopeptidase domain 17	rs29221466	6.87891E-07	Hepatic
Pdia6	12	protein disulfide isomerase associated 6	rs29129312	7.8158E-07	Hepatic

ASO Activity *trans*-eQTL Chromosome 10 rs29212236

Gene Symbol	Gene Chr.	Gene Name	rsID	P value	Localization
Nat8f4	6	N-acetyltransferase 8 (GCN5-related) family member 4	rs49246328	3.70932E-11	Hepatic
Phf20	2	PHD finger protein 20	rs31637355	1.62753E-10	Non-Hepatic
Akr1c12	13	aldo-keto reductase family 1, member C12	rs50447450	1.64658E-10	Hepatic
1700020D05Rik	19	RIKEN cDNA 1700020D05 gene	rs31634792	1.19748E-08	
Akr1c18	13	aldo-keto reductase family 1, member C18	rs51604584	1.24107E-08	Non-Hepatic
Isy1	6	ISY1 splicing factor homolog	rs47798216	1.59559E-08	Hepatic
Isoc2b	7	isochorismatase domain containing 2b	rs31633045	3.43383E-08	Hepatic
Plekhb2	1	pleckstrin homology domain containing, family B (evectins) member 2	rs3671264	2.365E-07	Hepatic
Nnt	13	nicotinamide nucleotide transhydrogenase	rs49246328	2.76016E-07	Hepatic
BC003331	1	cDNA sequence BC003331	rs31613420	3.18878E-07	Hepatic
Sh3gl1	17	SH3-domain GRB2-like 1	rs31638728	5.51104E-07	Hepatic
Csrp1	1	cysteine and glycine-rich protein 1	rs47244066	9.40926E-07	Non-Hepatic
Cdc23	18	CDC23 cell division cycle 23	rs29199437	1.03093E-06	Non-Hepatic
Qrsl1	10	glutaminyl-tRNA synthase (glutamine-hydrolyzing)-like 1	rs29207456	1.04028E-06	Hepatic
Wnt2	6	wingless-type MMTV integration site family, member 2	rs29223304	1.40902E-06	Hepatic
Cttnbp2nl	3	CTTNBP2 N-terminal like	rs29151504	2.46168E-06	Non-Hepatic
Pign	1	phosphatidylinositol glycan anchor biosynthesis, class N	rs29151504	2.47649E-06	Hepatic
Cdk8	5	cyclin-dependent kinase 8	rs29159678	2.59277E-06	Hepatic
Fggy	4	FGGY carbohydrate kinase domain containing	rs29159678	2.63189E-06	Hepatic
Rwdd3	3	RWD domain containing 3	rs29485521	2.70038E-06	Hepatic
Gdi2	13	guanosine diphosphate (GDP) dissociation inhibitor 2	rs48155658	2.81577E-06	Hepatic
Armcx4	X	armadillo repeat containing, X-linked 4	rs47798216	2.91107E-06	Non-Hepatic
Anxa5	3	annexin A5	rs31631407	2.99185E-06	Hepatic
Erbb2ip	13	Erbb2 interacting protein	rs29151504	3.00204E-06	Hepatic

Yae1d1	13	Yae1 domain containing 1	rs49694500	3.02897E-06	Hepatic
Fbxw2	2	F-box and WD-40 domain protein 2	rs47034083	3.02919E-06	Hepatic
Vopp1	6	vesicular, overexpressed in cancer, prosurvival protein 1	rs29160847	3.08097E-06	Non-Hepatic
Tiprl	1	TIP41, TOR signalling pathway regulator-like	rs49264506	3.14724E-06	Hepatic
Ttc3	16	tetratricopeptide repeat domain 3	rs48580986	3.79857E-06	Non-Hepatic
