# Supplementary material for: Mouse genome-wide association studies and systems genetics uncover the genetic architecture associated with hepatic pharmacokinetic and pharmacodynamic properties of a constrained ethyl antisense oligonucleotide targeting Malat1
Source: PLoS Genet. 2018 Oct 29;14(10):e1007732. doi: 10.1371/journal.pgen.1007732 (PMC6224167; doi:10.1371/journal.pgen.1007732)
Supplement: S6 Table — (PDF) [file pgen.1007732.s016.pdf]

**S6 Table**
**LD blocks surrounding SNPs Identified by GWA**

| rsID | snp_chr | snp_bp_mm10 | chr | start_bp_mm10 | end_bp_mm10 |
|---|---|---|---|---|---|
| rs27549337 | 4 | 126774007 | 4 | 124816685 | 124829647 |
| rs27549337 | 4 | 126774007 | 4 | 124831247 | 124853299 |
| rs27549337 | 4 | 126774007 | 4 | 124873534 | 124876272 |
| rs27549337 | 4 | 126774007 | 4 | 124888014 | 124952149 |
| rs27549337 | 4 | 126774007 | 4 | 124968416 | 125000112 |
| rs27549337 | 4 | 126774007 | 4 | 125027491 | 125188528 |
| rs27549337 | 4 | 126774007 | 4 | 125198655 | 125319261 |
| rs27549337 | 4 | 126774007 | 4 | 125328263 | 125364526 |
| rs27549337 | 4 | 126774007 | 4 | 125375619 | 125460155 |
| rs27549337 | 4 | 126774007 | 4 | 125497086 | 125502617 |
| rs27549337 | 4 | 126774007 | 4 | 125503937 | 125513441 |
| rs27549337 | 4 | 126774007 | 4 | 125516382 | 125521626 |
| rs27549337 | 4 | 126774007 | 4 | 125524998 | 125541656 |
| rs27549337 | 4 | 126774007 | 4 | 125548823 | 125549318 |
| rs27549337 | 4 | 126774007 | 4 | 125561631 | 125586719 |
| rs27549337 | 4 | 126774007 | 4 | 125594720 | 125595375 |
| rs27549337 | 4 | 126774007 | 4 | 125597951 | 125599891 |
| rs27549337 | 4 | 126774007 | 4 | 125612235 | 125620871 |
| rs27549337 | 4 | 126774007 | 4 | 125621738 | 125630258 |
| rs27549337 | 4 | 126774007 | 4 | 125649027 | 125663587 |
| rs27549337 | 4 | 126774007 | 4 | 125690023 | 125770764 |
| rs27549337 | 4 | 126774007 | 4 | 125788339 | 125789139 |
| rs27549337 | 4 | 126774007 | 4 | 125792013 | 125811187 |
| rs27549337 | 4 | 126774007 | 4 | 125826817 | 125880822 |
| rs27549337 | 4 | 126774007 | 4 | 125889238 | 125889269 |
| rs27549337 | 4 | 126774007 | 4 | 125899178 | 125927138 |
| rs27549337 | 4 | 126774007 | 4 | 125962758 | 125965118 |
| rs27549337 | 4 | 126774007 | 4 | 125971866 | 125975874 |
| rs27549337 | 4 | 126774007 | 4 | 126034140 | 126037913 |
| rs27549337 | 4 | 126774007 | 4 | 126039044 | 126273181 |
| rs27549337 | 4 | 126774007 | 4 | 126294351 | 126461456 |
| rs27549337 | 4 | 126774007 | 4 | 126472846 | 126477847 |
| rs27549337 | 4 | 126774007 | 4 | 126485839 | 126487690 |
| rs27549337 | 4 | 126774007 | 4 | 126488165 | 126671848 |
| rs27549337 | 4 | 126774007 | 4 | 126672146 | 126695146 |
| rs27549337 | 4 | 126774007 | 4 | 126696201 | 126712281 |
| rs27549337 | 4 | 126774007 | 4 | 126734251 | 127165470 |
| rs27549337 | 4 | 126774007 | 4 | 127228726 | 127280105 |
| rs27549337 | 4 | 126774007 | 4 | 127285214 | 127291260 |

| | | | | | |
|---|---|---|---|---|---|
| rs27549337 | 4 | 126774007 | 4 | 127299142 | 127300371 |
| rs27549337 | 4 | 126774007 | 4 | 127321972 | 127360080 |
| rs27549337 | 4 | 126774007 | 4 | 127366292 | 127391216 |
| rs27549337 | 4 | 126774007 | 4 | 127407210 | 127447944 |
| rs27549337 | 4 | 126774007 | 4 | 127452744 | 127480791 |
| rs27549337 | 4 | 126774007 | 4 | 127517708 | 127517967 |
| rs27549337 | 4 | 126774007 | 4 | 127539549 | 127717729 |
| rs27549337 | 4 | 126774007 | 4 | 127745162 | 127749711 |
| rs27549337 | 4 | 126774007 | 4 | 127755598 | 127783917 |
| rs27549337 | 4 | 126774007 | 4 | 127848768 | 127868230 |
| rs27549337 | 4 | 126774007 | 4 | 127905957 | 127921349 |
| rs27549337 | 4 | 126774007 | 4 | 127929098 | 127930609 |
| rs27549337 | 4 | 126774007 | 4 | 127938198 | 127959897 |
| rs27549337 | 4 | 126774007 | 4 | 127997215 | 127997269 |
| rs27549337 | 4 | 126774007 | 4 | 128003438 | 128009282 |
| rs27549337 | 4 | 126774007 | 4 | 128019499 | 128042169 |
| rs27549337 | 4 | 126774007 | 4 | 128043693 | 128095358 |
| rs27549337 | 4 | 126774007 | 4 | 128105098 | 128106764 |
| rs27549337 | 4 | 126774007 | 4 | 128106988 | 128114746 |
| rs27549337 | 4 | 126774007 | 4 | 128123155 | 128151199 |
| rs27549337 | 4 | 126774007 | 4 | 128151742 | 128160116 |
| rs27549337 | 4 | 126774007 | 4 | 128179201 | 128211382 |
| rs27549337 | 4 | 126774007 | 4 | 128228943 | 128253155 |
| rs27549337 | 4 | 126774007 | 4 | 128259871 | 128259896 |
| rs27549337 | 4 | 126774007 | 4 | 128263505 | 128265182 |
| rs27549337 | 4 | 126774007 | 4 | 128268268 | 128278689 |
| rs27549337 | 4 | 126774007 | 4 | 128281019 | 128281285 |
| rs27549337 | 4 | 126774007 | 4 | 128292007 | 128398302 |
| rs27549337 | 4 | 126774007 | 4 | 128412477 | 128414836 |
| rs27549337 | 4 | 126774007 | 4 | 128416038 | 128417120 |
| rs27549337 | 4 | 126774007 | 4 | 128421222 | 128522069 |
| rs27549337 | 4 | 126774007 | 4 | 128549273 | 128612509 |
| rs27549337 | 4 | 126774007 | 4 | 128613603 | 128613659 |
| rs27549337 | 4 | 126774007 | 4 | 128620719 | 128720391 |
| rs29210579 | 12 | 16857727 | 12 | 15103689 | 15153405 |
| rs29210579 | 12 | 16857727 | 12 | 15213407 | 15434657 |
| rs29210579 | 12 | 16857727 | 12 | 15490515 | 15578144 |
| rs29210579 | 12 | 16857727 | 12 | 15578336 | 15587512 |
| rs29210579 | 12 | 16857727 | 12 | 15604021 | 15616357 |
| rs29210579 | 12 | 16857727 | 12 | 15648467 | 15696710 |
| rs29210579 | 12 | 16857727 | 12 | 15720510 | 15801338 |
| rs29210579 | 12 | 16857727 | 12 | 15833645 | 15833759 |
| rs29210579 | 12 | 16857727 | 12 | 15833793 | 15854718 |

| | | | | | |
|---|---|---|---|---|---|
| rs29210579 | 12 | 16857727 | 12 | 15900811 | 15922258 |
| rs29210579 | 12 | 16857727 | 12 | 15954217 | 15984931 |
| rs29210579 | 12 | 16857727 | 12 | 15996968 | 16110069 |
| rs29210579 | 12 | 16857727 | 12 | 16120919 | 16123157 |
| rs29210579 | 12 | 16857727 | 12 | 16123355 | 16155099 |
| rs29210579 | 12 | 16857727 | 12 | 16162569 | 16316071 |
| rs29210579 | 12 | 16857727 | 12 | 16324110 | 16330020 |
| rs29210579 | 12 | 16857727 | 12 | 16359784 | 16362512 |
| rs29210579 | 12 | 16857727 | 12 | 16378994 | 16445681 |
| rs29210579 | 12 | 16857727 | 12 | 16448222 | 16472981 |
| rs29210579 | 12 | 16857727 | 12 | 16515492 | 16524211 |
| rs29210579 | 12 | 16857727 | 12 | 16529296 | 16535167 |
| rs29210579 | 12 | 16857727 | 12 | 16541381 | 16547503 |
| rs29210579 | 12 | 16857727 | 12 | 16557640 | 16564334 |
| rs29210579 | 12 | 16857727 | 12 | 16565189 | 16582244 |
| rs29210579 | 12 | 16857727 | 12 | 16582340 | 16593957 |
| rs29210579 | 12 | 16857727 | 12 | 16593983 | 16594457 |
| rs29210579 | 12 | 16857727 | 12 | 16598233 | 16645740 |
| rs29210579 | 12 | 16857727 | 12 | 16646101 | 16680271 |
| rs29210579 | 12 | 16857727 | 12 | 16687462 | 16711494 |
| rs29210579 | 12 | 16857727 | 12 | 16711530 | 16854837 |
| rs29210579 | 12 | 16857727 | 12 | 16857727 | 16860065 |
| rs29210579 | 12 | 16857727 | 12 | 16860626 | 17063981 |
| rs29210579 | 12 | 16857727 | 12 | 17070621 | 17082651 |
| rs29210579 | 12 | 16857727 | 12 | 17083055 | 17276156 |
| rs29210579 | 12 | 16857727 | 12 | 17283212 | 17285161 |
| rs29210579 | 12 | 16857727 | 12 | 17286624 | 17293729 |
| rs29210579 | 12 | 16857727 | 12 | 17293979 | 17296393 |
| rs29210579 | 12 | 16857727 | 12 | 17301821 | 17383504 |
| rs29210579 | 12 | 16857727 | 12 | 17384937 | 17387028 |
| rs29210579 | 12 | 16857727 | 12 | 17388152 | 17524367 |
| rs29210579 | 12 | 16857727 | 12 | 17528767 | 17530141 |
| rs29210579 | 12 | 16857727 | 12 | 17538582 | 17617582 |
| rs29210579 | 12 | 16857727 | 12 | 17617703 | 17653377 |
| rs29210579 | 12 | 16857727 | 12 | 17654025 | 17658126 |
| rs29212236 | 12 | 25280545 | 12 | 24748139 | 24779782 |
| rs29212236 | 12 | 25280545 | 12 | 24780083 | 24781859 |
| rs29212236 | 12 | 25280545 | 12 | 24782331 | 24782914 |
| rs29212236 | 12 | 25280545 | 12 | 24785580 | 24812100 |
| rs29212236 | 12 | 25280545 | 12 | 24815431 | 24897012 |
| rs29212236 | 12 | 25280545 | 12 | 24897457 | 24913655 |
| rs29212236 | 12 | 25280545 | 12 | 24917304 | 24917463 |
| rs29212236 | 12 | 25280545 | 12 | 24918998 | 24944427 |

| | | | | | |
|---|---|---|---|---|---|
| rs29212236 | 12 | 25280545 | 12 | 24970457 | 25040229 |
| rs29212236 | 12 | 25280545 | 12 | 25041573 | 25059932 |
| rs29212236 | 12 | 25280545 | 12 | 25070248 | 25070986 |
| rs29212236 | 12 | 25280545 | 12 | 25108680 | 25178260 |
| rs29212236 | 12 | 25280545 | 12 | 25181881 | 25189755 |
| rs29212236 | 12 | 25280545 | 12 | 25191454 | 25194228 |
| rs29212236 | 12 | 25280545 | 12 | 25195354 | 25195619 |
| rs29212236 | 12 | 25280545 | 12 | 25196017 | 25207402 |
| rs29212236 | 12 | 25280545 | 12 | 25224744 | 25226732 |
| rs29212236 | 12 | 25280545 | 12 | 25230697 | 25275939 |
| rs29212236 | 12 | 25280545 | 12 | 25277307 | 25279910 |
| rs29212236 | 12 | 25280545 | 12 | 25280545 | 25310309 |
| rs29212236 | 12 | 25280545 | 12 | 25318580 | 25319015 |
| rs29212236 | 12 | 25280545 | 12 | 25322466 | 25358466 |
| rs29212236 | 12 | 25280545 | 12 | 25361339 | 25361877 |
| rs29212236 | 12 | 25280545 | 12 | 25373356 | 25406768 |
| rs29212236 | 12 | 25280545 | 12 | 25442631 | 25443390 |
| rs29212236 | 12 | 25280545 | 12 | 25447183 | 25488985 |
| rs29212236 | 12 | 25280545 | 12 | 25493838 | 25497295 |
| rs29212236 | 12 | 25280545 | 12 | 25499223 | 25499814 |
| rs29212236 | 12 | 25280545 | 12 | 25500348 | 25527117 |
| rs29212236 | 12 | 25280545 | 12 | 25533313 | 25534838 |
| rs29212236 | 12 | 25280545 | 12 | 25535511 | 25559076 |
| rs29212236 | 12 | 25280545 | 12 | 25563107 | 25584889 |
| rs29212236 | 12 | 25280545 | 12 | 25590322 | 25596690 |
| rs29212236 | 12 | 25280545 | 12 | 25607034 | 25632211 |
| rs29212236 | 12 | 25280545 | 12 | 25632394 | 25654701 |
| rs29212236 | 12 | 25280545 | 12 | 25666182 | 25676139 |
| rs29212236 | 12 | 25280545 | 12 | 25682165 | 25791228 |
| rs29212236 | 12 | 25280545 | 12 | 25806530 | 26189890 |
| rs29212236 | 12 | 25280545 | 12 | 26190902 | 26224856 |
| rs29212236 | 12 | 25280545 | 12 | 26229769 | 26814216 |
| rs29212236 | 12 | 25280545 | 12 | 26815428 | 26853836 |
| rs29212236 | 12 | 25280545 | 12 | 26854028 | 26896849 |
| rs29212236 | 12 | 25280545 | 12 | 26904978 | 26919404 |
| rs29364476 | 10 | 87351304 | 10 | 85443007 | 85578860 |
| rs29364476 | 10 | 87351304 | 10 | 85588430 | 85590449 |
| rs29364476 | 10 | 87351304 | 10 | 85592697 | 85644817 |
| rs29364476 | 10 | 87351304 | 10 | 85668865 | 85671988 |
| rs29364476 | 10 | 87351304 | 10 | 85695224 | 86052302 |
| rs29364476 | 10 | 87351304 | 10 | 86069728 | 86096474 |
| rs29364476 | 10 | 87351304 | 10 | 86106726 | 86462590 |
| rs29364476 | 10 | 87351304 | 10 | 86496115 | 86724189 |

| | | | | | |
|---|---|---|---|---|---|
| rs29364476 | 10 | 87351304 | 10 | 86733809 | 86733857 |
| rs29364476 | 10 | 87351304 | 10 | 86734259 | 87097360 |
| rs29364476 | 10 | 87351304 | 10 | 87100880 | 87184782 |
| rs29364476 | 10 | 87351304 | 10 | 87193299 | 87204155 |
| rs29364476 | 10 | 87351304 | 10 | 87299947 | 87339419 |
| rs29364476 | 10 | 87351304 | 10 | 87355439 | 87506187 |
| rs29364476 | 10 | 87351304 | 10 | 87527763 | 88217182 |
| rs29364476 | 10 | 87351304 | 10 | 88217702 | 88223398 |
| rs29364476 | 10 | 87351304 | 10 | 88230477 | 88586985 |
| rs29364476 | 10 | 87351304 | 10 | 88601134 | 88705281 |
| rs29364476 | 10 | 87351304 | 10 | 88715806 | 88734069 |
| rs29364476 | 10 | 87351304 | 10 | 88734106 | 88972342 |
| rs29364476 | 10 | 87351304 | 10 | 88972723 | 88972872 |
| rs29364476 | 10 | 87351304 | 10 | 89061138 | 89117012 |
| rs29364476 | 10 | 87351304 | 10 | 89134881 | 89183688 |
| rs29364476 | 10 | 87351304 | 10 | 89189549 | 89230599 |
| rs29364476 | 10 | 87351304 | 10 | 89265990 | 89272261 |
| rs32062485 | 4 | 153939301 | 4 | 151977977 | 152739036 |
| rs32062485 | 4 | 153939301 | 4 | 152802427 | 153800508 |
| rs32062485 | 4 | 153939301 | 4 | 153842656 | 153914688 |
| rs32062485 | 4 | 153939301 | 4 | 153933969 | 154072413 |
| rs32062485 | 4 | 153939301 | 4 | 154099743 | 154137684 |
| rs32062485 | 4 | 153939301 | 4 | 154178101 | 154359380 |
| rs32062485 | 4 | 153939301 | 4 | 154361065 | 154493779 |
| rs32062485 | 4 | 153939301 | 4 | 154505695 | 154509428 |
| rs32062485 | 4 | 153939301 | 4 | 154526989 | 154539849 |
| rs32062485 | 4 | 153939301 | 4 | 154540376 | 154552257 |
| rs32062485 | 4 | 153939301 | 4 | 154575679 | 154746734 |
| rs32062485 | 4 | 153939301 | 4 | 154750253 | 154758258 |
| rs32062485 | 4 | 153939301 | 4 | 154758394 | 154784147 |
| rs32062485 | 4 | 153939301 | 4 | 154799715 | 154821693 |
| rs32062485 | 4 | 153939301 | 4 | 154823258 | 154828174 |
| rs32062485 | 4 | 153939301 | 4 | 154828856 | 154867926 |
| rs32062485 | 4 | 153939301 | 4 | 154870736 | 154883306 |
| rs32062485 | 4 | 153939301 | 4 | 154914410 | 155184127 |
| rs32062485 | 4 | 153939301 | 4 | 155210788 | 155234223 |
| rs32062485 | 4 | 153939301 | 4 | 155376270 | 155430194 |
| rs32062485 | 4 | 153939301 | 4 | 155454211 | 155456179 |
| rs32062485 | 4 | 153939301 | 4 | 155511694 | 155567444 |
| rs32062485 | 4 | 153939301 | 4 | 155610933 | 155620808 |
| rs32062485 | 4 | 153939301 | 4 | 155628936 | 155910817 |
